# *Haemagogus leucocelaenus* and *Haemagogus janthinomys* are the primary vectors in the major yellow fever outbreak in Brazil, 2016–2018

**DOI:** 10.1080/22221751.2019.1568180

**Published:** 2019-02-01

**Authors:** Filipe Vieira Santos de Abreu, Ieda Pereira Ribeiro, Anielly Ferreira-de-Brito, Alexandre Araujo Cunha dos Santos, Rafaella Moraes de Miranda, Iule de Souza Bonelly, Maycon Sebastião Alberto Santos Neves, Maria Ignez Bersot, Taissa Pereira dos Santos, Marcelo Quintela Gomes, José Luis da Silva, Alessandro Pecego Martins Romano, Roberta Gomes Carvalho, Rodrigo Fabiano do Carmo Said, Mario Sergio Ribeiro, Roberto da Costa Laperrière, Eduardo Oyama Lins Fonseca, Aloísio Falqueto, Christophe Paupy, Anna-Bella Failloux, Sara Moutailler, Marcia Gonçalves de Castro, Mariela Martínez Gómez, Monique de Albuquerque Motta, Myrna Cristina Bonaldo, Ricardo Lourenço-de-Oliveira

**Affiliations:** a Laboratório de Mosquitos Transmissores de Hematozoários, Instituto Oswaldo Cruz, Rio de Janeiro, RJ, Brazil; b Instituto Federal do Norte de Minas Gerais, Salinas, MG, Brazil; c Laboratório de Biologia Molecular de Flavivírus, Instituto Oswaldo Cruz, Rio de Janeiro, RJ, Brazil; d MIVEGEC Laboratory, IRD-CNRS Université de Montpellier, Montpellier, France; e Gerência de Estudos e Pesquisas em Antropozoonoses, Secretaria Estadual de Saúde, Rio de Janeiro, RJ, Brazil; f Coordenação Geral de Vigilância das Doenças Transmissíveis, Departamento de Vigilância das Doenças Transmissíveis, Secretaria de Vigilância em Saúde, Ministério da Saúde, Brasília, DF, Brazil; g Departamento de Saúde Ambiental e Saúde do Trabalhador, Secretaria de Vigilância em Saúde, Ministério da Saúde, Brasília, DF, Brazil; h Subsecretaria de Vigilância e Proteção à Saúde de Minas Gerais, Belo Horizonte, MG, Brazil; i Superintendência de Vigilância Epidemiológica e Ambiental, Secretaria Estadual de Saúde, Rio de Janeiro, RJ, Brazil; j Núcleo Especial de Vigilância Ambiental, Secretaria Estadual de Saúde do Espírito Santo, Vitória, ES, Brazil; k Secretaria de Saúde do Estado da Bahia, Salvador, Bahia, Brazil; l Universidade Federal do Espírito Santo, Vitória, ES, Brazil; m Arboviruses and Insect Vectors, Institut Pasteur, Paris, France; n UMR BIPAR, Animal Health Laboratory, ANSES, INRA, Ecole Nationale Vétérinaire d'Alfort, Université Paris-Est, Maisons-Alfort, France

**Keywords:** Yellow fever, Atlantic forest, *Haemagogus*, *Aedes*, *Sabethes*

## Abstract

The yellow fever virus (YFV) caused a severe outbreak in Brazil in 2016–2018 that rapidly spread across the Atlantic Forest in its most populated region without viral circulation for almost 80 years. A comprehensive entomological survey combining analysis of distribution, abundance and YFV natural infection in mosquitoes captured before and during the outbreak was conducted in 44 municipalities of five Brazilian states. In total, 17,662 mosquitoes of 89 species were collected. Before evidence of virus circulation, mosquitoes were tested negative but traditional vectors were alarmingly detected in 82% of municipalities, revealing high receptivity to sylvatic transmission. During the outbreak, five species were found positive in 42% of municipalities. *Haemagogus janthinomys* and *Hg. leucocelaenus* are considered the primary vectors due to their large distribution combined with high abundance and natural infection rates, concurring together for the rapid spread and severity of this outbreak. *Aedes taeniorhynchus* was found infected for the first time, but like *Sabethes chloropterus* and *Aedes scapularis*, it appears to have a potential local or secondary role because of their low abundance, distribution and infection rates. There was no evidence of YFV transmission by *Aedes albopictus* and *Aedes aegypti,* although the former was the most widespread species across affected municipalities, presenting an important overlap between the niches of the sylvatic vectors and the anthropic ones. The definition of receptive areas, expansion of vaccination in the most affected age group and exposed populations and the adoption of universal vaccination to the entire Brazilian population need to be urgently implemented.

## Introduction

Yellow fever (YF) is a viral disease that decimated populations and harmed commercial routes in the Americas in the nineteenth century and continues to induce a heavy public health burden by annually causing thousands of cases and deaths in Africa and South America despite the existence of effective vaccines [1,2]. The etiological agent of this disease is the yellow fever virus (YFV), which has originated in Africa and spread to the Americas and the Caribbean probably during the seventeenth—nineteenth centuries. The discovery that YFV is transmitted by the bite of the domestic mosquito *Aedes* (*Stegomyia*) *aegypti* revolutionized the understanding of its epidemiology and guided control and protection measures for urban human populations early in the twentieth century [3–5].

Since the 1930s, two main YFV transmission cycles have been described: the sylvatic, in which the virus is transmitted by arboreal mosquitoes between non-human primates (NHP) in the forest and where humans can be incidentally infected [6–8] and the urban, maintained between *Ae. aegypti* and humans [9]. An intermediate/rural cycle has been so far described only in Africa [10]. While the transmitters in the sylvatic and intermediate cycles in Africa are *Aedes* mosquitoes of the subgenera *Stegomyia* and *Diceromyia*, this role is played by species of genus *Haemagogus* and *Sabethes* in the New World*,* considered the primary and secondary sylvatic vectors respectively [10]. If the elimination of the urban cycle is feasible as it has occurred very rarely in the Americas since 1942, the enzootic sylvatic one is considered ineradicable [8,11]. The sylvatic cycle consists of a permanent threat both for its spillover to an urban cycle in the nearby of highly *Ae. aegypti* infested locals as well as for the emergency of epidemics where vaccination coverage in risk areas is inadequate [11,12]. This was the case of the severe outbreak recorded in southeastern Brazil in 2016–2018 [11,13].

Intriguingly, no record of YFV circulation had been detected since the 1930s in the Atlantic Forest zone in Brazil, the biome where sylvatic transmission was first discovered [14,15]. Hence, in contrast with the perennial transmission focus represented by the endemic/enzootic Amazon region and epizootic/YFV emerging areas in the *Cerrado* biome, the Brazilian health authorities have excluded this east-coastal zone from the YFV national vaccination program for decades [9,11,12]. Between the mid-twentieth century and 1999, YFV expansion and retraction waves originated in the Amazon have spread southward across the *Cerrado*, but extra-Amazon epizootics and epidemics were essentially limited to the Central-West region. However, from 2000 on, YFV expansion waves have reached the pampa biome in the southernmost Brazilian state and progressively spread eastward across the *Cerrado*. In late 2016, it spilled over from the *Cerrado* into transition zone between this biome and the Atlantic Forest in Minas Gerais state (MG) and rapidly spread across this last biome in the southeast. This region records the highest population densities in the country, but vaccination coverage against YFV was almost null at that moment. Then, the country’s largest outbreak of sylvatic YF erupted, and rapidly spread in the southeast states of MG, Espírito Santo (ES), Rio de Janeiro (RJ) and São Paulo (SP) [11,16]. In less than two years, it has caused 2,058 confirmed human cases and 689 deaths, rates not observed since the first half of the twentieth century. It also caused a huge impact on NHP biodiversity as consequence of thousands of epizootic events [17,18]. The outbreak spread more than 900 km at an estimated speed of around 3 km a day [13,19]. The movement of paucisymptomatic and asymptomatic viremic humans and displacement of infected mosquitoes has been suggested as the main factors inducing this rapid spatial spread [11]. Therefore, defining the main vectors involved in the sylvatic transmission is critical in understanding the main ecological risk factors driving this unprecedented outbreak and guiding public health measures.

We conducted a comprehensive entomological survey based on a combined analysis of distribution, abundance and YFV natural infections in mosquitoes before and during the outbreak in the Brazilian states affected by this sanitary disaster in order to determine the primary vectors in the sylvatic cycle, and clarify the role of anthropic and domestic mosquitoes such as *Ae. (Stegomyia) albopictus* and *Ae. aegypti*.

## Results

In total, 17,662 mosquitoes (15,398 adults and 2,264 immatures) belonging to 89 species were collected (Table 1).

**Table 1.** Mosquitoes species in decreasing order of adults collected before and during the YFV outbreak, from May 2015 to May 2018, in 44 municipalities of four Southeast Brazil states and Bahiaian in the Northeast: We also present life stage, number of pool tested, number of positive pools, infection rates and percentage of presence in the sampled municipalities.

	No Adult. before	No Adult. during	No Total adult.	Relative abundance (%)^1^	No of immature	No Mosquito total	Pools tested^2^ (Positives)	% of tested pools	MIR^3^	MLE^4^	Pres. bef.^5^(%) *n* = 28	Pres. dur.^6^(%) *n* = 21
*Ae. scapularis*	1096	1870	2966	19.262	0	2966	403(1)	72.9	0.54	0.54	67.9	85.7
*Ae. taeniorhynchus*	219	2428	2647	17.191	0	2647	199(1)	67.5	0.59	0.59	7.1	23.8
*Hg. leucocelaenus*	525	895	1420	9.222	1419	2839	327(41)	83.2	34.92	37.65	78.5	71.4
*Ae. albopictus*	329	478	807	5.241	439	1246	262(0)	87.6	0	0	57.1	95.2
*Hg. janthinomys*	89	527	616	4.001	27	643	162(20)	94.2	34.48	36.35	39.2	57.1
*Li. durhamii*	131	384	515	3.345	3	518	118(0)	63.4	0	0	60.7	76.2
*Sa. albiprivus*	132	302	434	2.819	42	476	173(0)	92.0	0	0	28.6	42.9
*Ps. ferox*	180	122	302	1.961	0	302	60(0)	69.8	0	0	42.9	57.1
*Sh. fluviatilis*	171	33	204	1.325	0	204	27(0)	73.0	0	0	35.7	14.3
*Wy. confusa*	71	121	192	1.247	0	192	41(0)	56.9	0	0	25.0	38.1
*Sa. petrocchiae'*	0	178	178	1.156	0	178	17(0)	39.5	0	0	0.0	4.8
*Ae. serratus*	67	73	140	0.909	0	140	37(0)	84.1	0	0	21.4	42.9
*Wy. pilicauda*	90	41	131	0.851	0	131	19(0)	59.4	0	0	39.3	23.8
*Ae. aegypti*	52	61	113	0.734	0	113	30(0)	75.0	0	0	10.7	28.6
*Ru. humboldti*	98	12	110	0.714	0	110	23(0)	82.1	0	0	25.0	19.0
*Wy. aporonoma/staminifera*	26	83	109	0.708	0	109	29(0)	53.7	0	0	35.7	52.4
*Ae. terrens*	77	26	103	0.669	128	231	30(0)	83.3	0	0	35.7	38.1
*On. personatum*	79	18	97	0.630	8	105	20(0)	71.4	0	0	21.4	28.6
*Ps. albipes*	92	1	93	0.604	0	93	10(0)	76.9	0	0	3.6	4.8
*Tr. pallidiventer*	57	36	93	0.604	0	93	32(0)	72.7	0	0	42.9	38.1
*Ru. frontosa*	70	17	87	0.565	0	87	20(0)	69.0	0	0	39.3	23.8
*Ma. indubitans*	1	81	82	0.533	0	82	13(0)	81.3	0	0	3.6	4.8
*Li. pseudomethisticus*	59	16	75	0.487	0	75	16(0)	69.6	0	0	21.4	23.8
*Wy. palmata/galvaoi*	35	24	59	0.383	0	59	10(0)	66.7	0	0	17.9	14.3
*Sa. fabricii/undosus*	9	46	55	0.357	0	55	19(0)	79.2	0	0	10.7	28.6
*Wy. medioalbipes*	12	41	53	0.344	0	53	15(0)	55.6	0	0	3.6	23.8
*Tr. digitatum*	36	14	50	0.325	0	50	17(0)	65.4	0	0	25.0	33.3
*Wy. mystes*	11	32	43	0.279	0	43	18(0)	62.1	0	0	17.9	38.1
*Sa. chloropterus*	11	31	42	0.273	0	42	21(1)	100.0	23.8	23.2	10.7	28.6
*Cx. quinquefasciatus*	9	31	40	0.260	0	40	3(0)	23.1	0	0	7.1	19.0
*Sa. aurescens*	31	7	38	0.247	12	50	12(0)	85.7	0	0	28.6	9.5
*Wy. davisi*	17	13	30	0.195	0	30	6(0)	75.0	0	0	17.9	4.8
*Ma. titillans*	9	20	29	0.188	0	29	3(0)	33.3	0	0	7.1	14.3
*Wy. bonnei/deanei*	18	7	25	0.162	0	25	7(0)	70.0	0	0	10.7	14.3
*Sa. melanonymphe*	17	5	22	0.143	7	29	13(0)	100.0	0	0	17.9	23.8
*Ru. cerqueirai*	16	5	21	0.136	0	21	5(0)	55.6	0	0	10.7	14.3
*Tr. castroi/similis*	20	1	21	0.136	0	21	6(0)	85.7	0	0	14.3	4.8
*Wy. incaudata*	11	10	21	0.136	0	21	7(0)	70.0	0	0	10.7	14.3
*Wy. edwardsi*	15	5	20	0.130	0	20	5(0)	55.6	0	0	14.3	14.3
*Cq. juxtamansonia*	0	17	17	0.110	0	17	6(0)	85.7	0	0	0.0	14.3
*Wy. bourrouli/forcipenis*	3	14	17	0.110	0	17	8(0)	61.5	0	0	7.1	23.8
*Wy. lutzi*	5	12	17	0.110	6	23	4(0)	30.8	0	0	14.3	19.0
*Sa. identicus*	6	10	16	0.104	1	17	10(0)	76.9	0	0	17.9	28.6
*Tr. compressum*	2	14	16	0.104	0	16	1(0)	8.3	0	0	7.1	14.3
*Ae. fluviatilis*	4	10	14	0.091	9	23	6(0)	100.0	0	0	10.7	9.5
*Cq. venezuelensis*	3	10	13	0.084	0	13	5(0)	50.0	0	0	7.1	9.5
*Sa. intermedius*	8	3	11	0.071	0	11	6(0)	85.7	0	0	14.3	9.5
*Ru. reversa/theobaldi*	10	0	10	0.065	0	10	2(0)	50.0	0	0	14.3	0.0
*Cq. nigricans*	0	8	8	0.052	0	8	1(0)	50.0	0	0	0.0	4.8
*Ps. lutzii/amazonica*	2	6	8	0.052	0	8	5(0)	100.0	0	0	3.6	9.5
*Sa. purpureus'*	1	7	8	0.052	0	8	3(0)	100.0	0	0	3.6	9.5
*Li. flavisetosus*	0	7	7	0.045	0	7	0(0)	0.0	0	0	0.0	14.3
*Ae. fulvithorax*	4	2	6	0.039	0	6	3(0)	60.0	0	0	10.7	4.8
*Wy. pallidoventer*	6	0	6	0.039	0	6	2(0)	100.0	0	0	3.6	0.0
*Cq. albicosta*	0	5	5	0.032	0	5	3(0)	100.0	0	0	0.0	9.5
*Sa. soperi*	2	3	5	0.032	0	5	4(0)	80.0	0	0	7.1	9.5
*Sa. whitmani*	0	5	5	0.032	0	5	2(0)	66.7	0	0	0.0	9.5
*Sa. xyphydes*	2	3	5	0.032	0	5	4(0)	100.0	0	0	3.6	14.3
*Wy. dyari*	0	5	5	0.032	0	5	1(0)	50.0	0	0	0.0	9.5
*Ae. condolences'*	0	4	4	0.026	0	4	1(0)	100.0	0	0	0.0	4.8
*Ae. rhyacophilus*	0	4	4	0.026	0	4	2(0)	50.0	0	0	0.0	14.3
*An. fluminensis*	4	0	4	0.026	0	4	0	0.0	0	0	3.6	0.0
*Wy. theobaldi*	4	0	4	0.026	0	4	2(0)	100.0	0	0	7.1	0.0
*Wy. antunesi*	3	0	3	0.019	0	3	2(0)	100.0	0	0	3.6	0.0
*Wy. oblita*	2	1	3	0.019	26	29	1(0)	50.0	0	0	7.1	4.8
*Ae. argyrothorax*	0	2	2	0.013	0	2	2(0)	100.0	0	0	0.0	9.5
*Tr. soaresi*	0	2	2	0.013	0	2	0	0.0	0	0	0.0	9.5
*Wy. codiocampa*	0	2	2	0.013	0	2	2(0)	100.0	0	0	3.6	0.0
*Wy. longirostris*	2	0	2	0.013	0	2	2(0)	100.0	0	0	7.1	0.0
*Wy. melanocephala*	1	1	2	0.013	0	2	2(0)	100.0	0	0	3.6	4.8
*Cq. hermanoi'*	0	1	1	0.006	0	1	1(0)	100.0	0	0	0.0	4.8
*Cq. shannoni*	0	1	1	0.006	0	1	1(0)	100.0	0	0	0.0	4.8
*Ps. pseudomelanota'*	1	0	1	0.006	0	1	0	0.0	0	0	3.6	0.0
*Sa. quasicyaneus*	0	1	1	0.006	0	1	1(0)	100.0	0	0	0.0	4.8
*Wy. arthrostigma'*	0	1	1	0.006	0	1	0	0.0	0	0	0.0	4.8
*Wy. cerqueirai*	1	0	1	0.006	0	1	1(0)	100.0	0	0	0.0	4.8
*Wy. exallos'*	1	0	1	0.006	0	1	1(0)	100.0	0	0	3.6	0.0
*Wy. knabi'*	0	1	1	0.006	0	1	1(0)	100.0	0	0	0.0	4.8
*Wy. shannoni*	0	1	1	0.006	0	1	0	0.0	0	0	0.0	4.8
*Other taxa*^7^	1276	1799	3075	19.970	137	3212	405 (0)	55.5	0	0	–	–
*Total*	5341	10057	15398	100	2264	17662	2738(64)	71.3	–	–	–	–

^1^Relative abundance is calculated by dividing the number of adults of one species by the number of adults of all species × 100. ^2^Number of adult pools tested; ^3^Minimum Infection Rate = No of positive pools/No of same species adults analyzed × 1000; ^4^Maximum Likelihood Estimate per 1000 mosquitoes = 1− (1−*Y*/*X*)^1/m^ where *Y* is the number of positive pools, *X* is the total number of pools, and *m* is the size of each tested pool; ^5^Percentual of municipalities where each species was found before the outbreak; ^6^Percentage of municipalities where each species was found during the outbreak. ^7^Other taxa were represented by: *Culex sp.; Wyeomyia sp.; An. cruzii; Cx. nigripalpus; Sabethes sp.; Runchomyia sp.; Aedes sp.; Coquillettidia sp.; Limatus sp.; Psorophora sp.; Anopheles sp.; Shannoniana sp.; Mansonia sp.; Trichoprosopon sp.; Cx. declarator’; Aedeomyia sp.; Lutzia sp.’; Culex coronator’; An. bellator; An. lutzi; An. mediopunctatus; An. neivai; Haemagogus sp*.

We marked with ‘ those taxa with ambiguous classification, due to the existence of complex of cryptic species.

**Figure 1.**
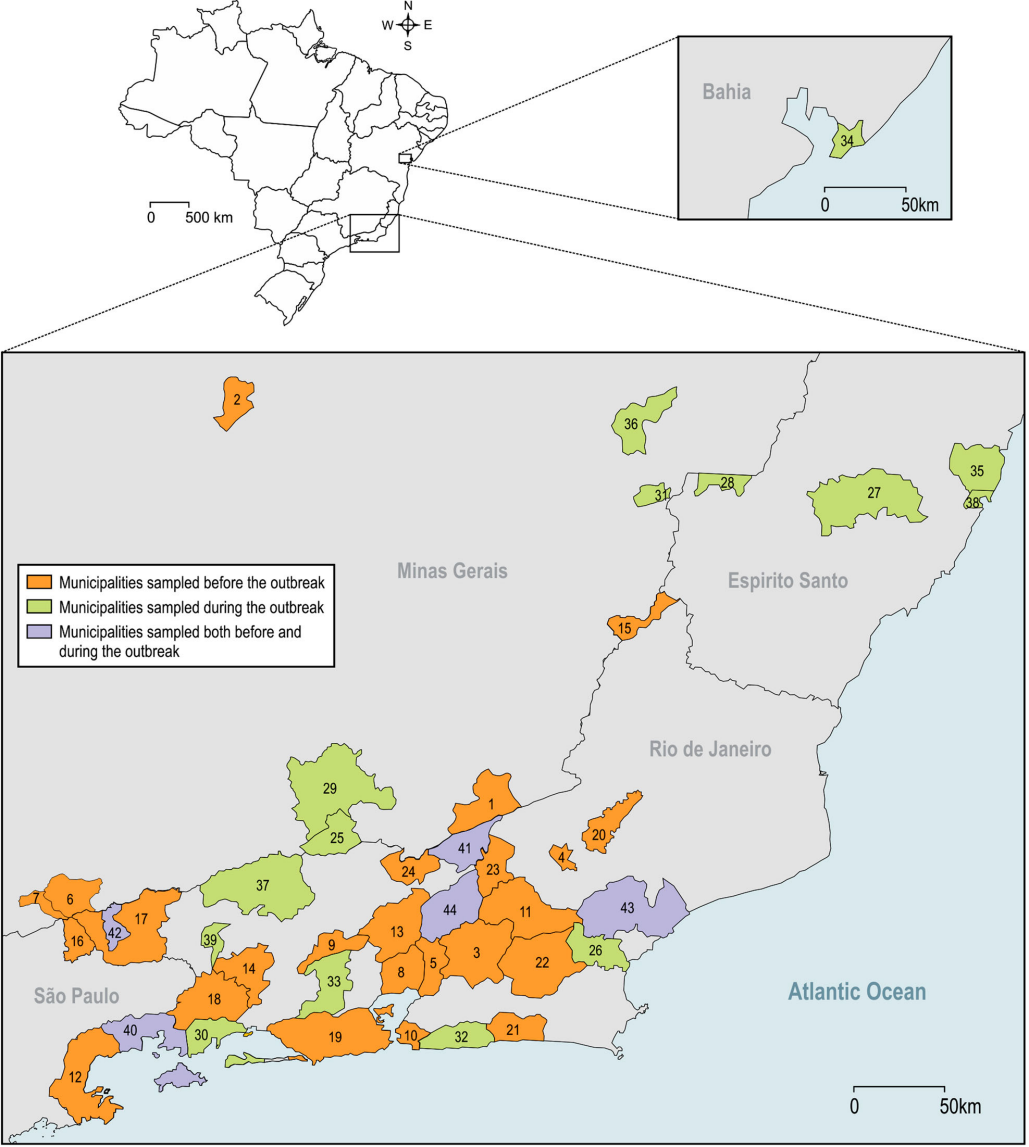
Brazilian municipalities sampled before, during and both before and during local YFV transmission. 1 – Além Paraíba; 2 – Belo Horizonte; 3 – Cachoeiras de Macacu; 4 – Cordeiros; 5 – Guapimirim; 6 – Itamonte; 7 – Itanhandu; 8 – Magé; 9 – Miguel Pereira; 10 – Niterói; 11 – Nova Friburgo; 12 – Paraty; 13 – Petrópolis; 14 – Piraí; 15 – Porciúncula; 16 – Queluz; 17 – Resende; 18 – Rio Claro; 19 – Rio de Janeiro; 20 – Silva Jardim; 21 – São Sebastião do Alto; 22 – Saquarema; 23 – Sumidouro; 24 – Três Rios; 25 – Belmiro Braga; 26 – Casimiro de Abreu; 27 – Domingos Martins; 28 – Ibatiba; 29 – Juiz de Fora; 30 – Mangaratiba; 31 – Manhumirim; 32 – Maricá; 33 – Nova Iguaçu; 34 – Salvador; 35 – Serra; 36 – Simonésia; 37 – Valença; 38 – Vitória; 39 – Volta Redonda; 40 – Angra dos reis; 41 – Carmo; 42 – Itatiaia; 43 – Macaé; 44 – Teresópolis.

**Figure 2.**
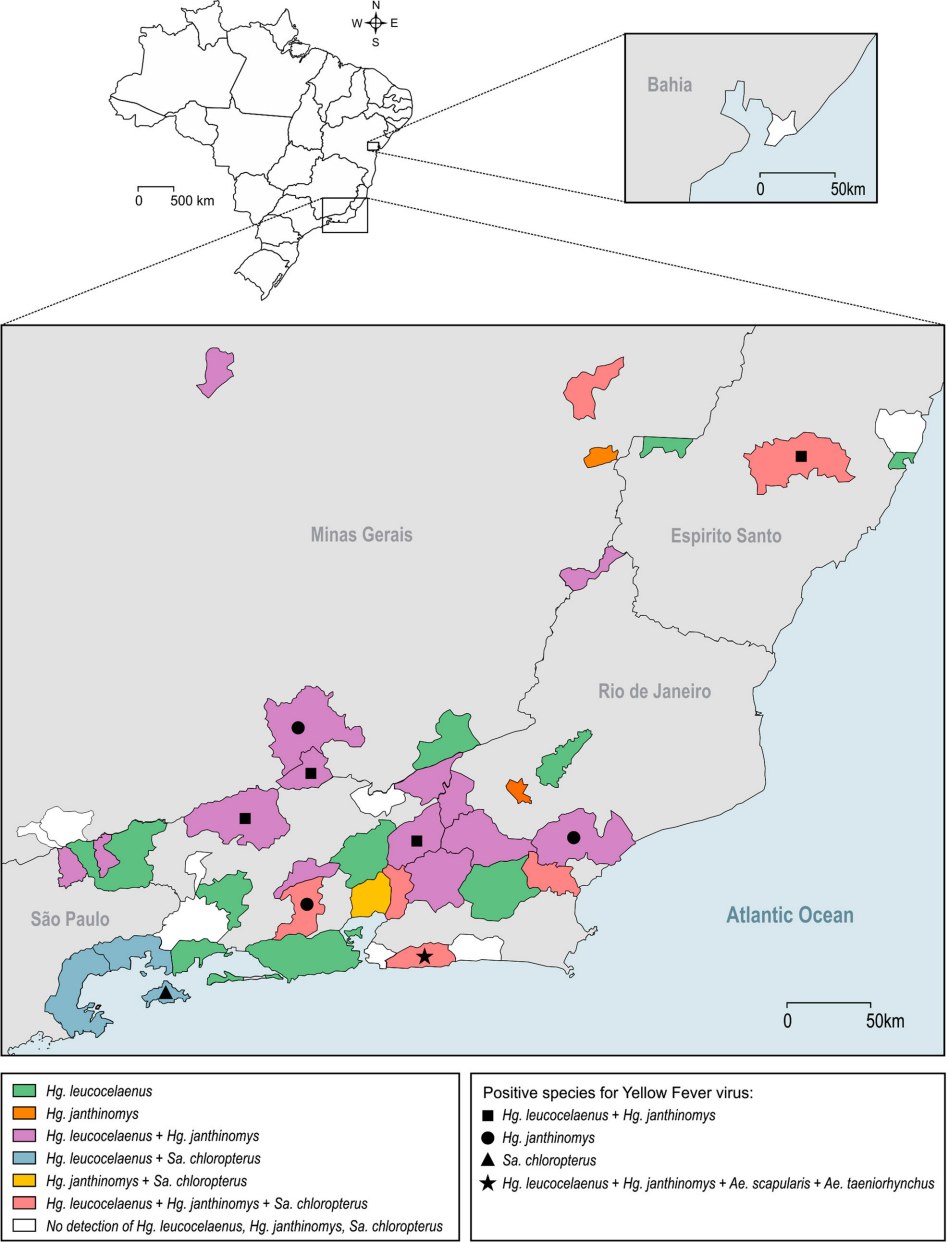
*Haemagogus leucocelaenus*, *Hg. janthinomys* and *Sabethes chloropterus* distribution along sampled municipalities. Geometric forms represented species found positive for yellow fever virus per municipality.

*Collections before the yellow fever outbreak:* During this period, 5,341 mosquitoes were collected in 28 municipalities in RJ and in two bordering states (SP and MG). *Haemagogus leucocelaenus* was the most widespread species, being detected in 78.5% of sampled municipalities. In addition, it was the second most abundant species, accounting for 9.8% of total adult caught mosquitoes. Regarding other traditional YFV vectors, *Hg. janthinomys* and *Sabethes chloropterus* were detected in 39.2% and 10.7% of the municipalities surveyed before virus circulation, with a relative abundance of 1.6% and 0.21%, respectively (Figures 1 and 2, Table 1 and S1).

In addition, *Aedes scapularis* and *Ae. albopictus* were respectively the second and fourth most widespread species, present in 67.9% and 57.1% of surveyed municipalities prior the outbreak. While *Ae. scapularis* was the most abundant species (20.6%), *Ae. albopictus* accounted for 6.1% of abundance (Table 1 and S1). Remarkably, *Psorophora ferox* and *Sabethes albiprivus* were among the most captured mosquitoes prior the outbreak (Table 1), with relative abundance >2%, being detected in 42.9 and 28.6% of sampled municipalities, respectively.

**Figure 3.**
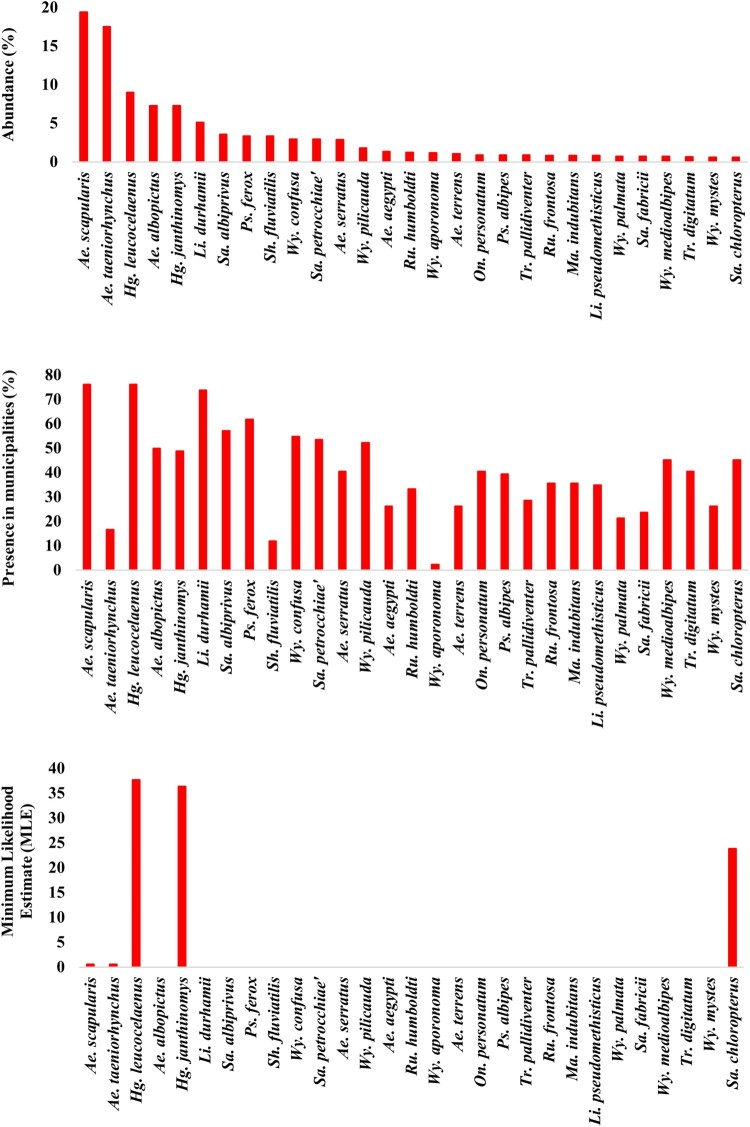
Percentage of abundance (1st graph) and presence (2nd graph) of the 29 most abundant species considering both before (between may/2015 and Jan/2017) and during YFV outbreak (between fev/2017 and may/2018) moments. 3rd graph shows the Maximum Likelihood Estimate (MLE) from the same species. Name of cryptic species e.g. *Hg. janthinomys*, *Wy. aporonoma/staminifera*, *Wy. palmata/galvaoi*, *Sa. fabricii/undosus* are abbreviated.

*Collections during the yellow fever outbreak:* In this epidemiological context, 10,057 mosquitoes were collected in 21 municipalities, five of which (20%) had also been sampled before YFV transmission. Remarkably, the density and abundance of *Hg. janthinomys* tripled in relation to the pre-epidemic period, while that of *Hg. leucocelaenus* continued to be high (Table 1, S1 ad S2). These *Haemagogus* species were detected in 57% and 71% of municipalities with local active YFV transmission, respectively. Intriguingly, *Ae. albopictus* was the most widespread (present in 95% of municipalities) and the fifth most abundant species (relative abundance = 4.75%) during the outbreak (Tables 1 and S1). Although *Ae. taeniorhynchus* was the most abundant species in the total collections made during the outbreak (24.1%), its distribution was restricted to five coastal lowland municipalities under influence of the Atlantic Forest biome. *Sabethes albiprivus* was the most widespread and abundant species of the genus, while *Sa. petrocchiae* although abundant, was collected in only one affected municipality (Simonésia – MG) located in the transition between the Atlantic Forest and *Cerrado* biomes (Tables 1 and S2, Figure 3).

*Virus detection:* RNA of 2,738 pools, containing 10,537 adult mosquitoes from 85 species, was extracted and tested for YFV. Virus genome was detected in 64 pools (2.3%) containing 323 mosquitoes belonging to five species: *Hg. janthinomys*, *Hg. leucocelaenus, Sa. chloropterus, Ae. scapularis* and *Ae. taeniorhynchus* (Tables 1 and 2). Positive mosquitoes were detected in 42.8% of surveyed municipalities (9/21) in three Brazilian states: 6 out of 17 from RJ (59 positive pools), two out of five from MG (3 positive pools) and one out of four from ES (2 positive pools) (Tables 2 and S3). Positive pools were found in mosquitoes captured in 2017 and 2018. No positive mosquito was found prior the detection of signals of YFV circulation in the respective municipalities. Also, YFV was not found in any pool of mosquitoes caught in Salvador (BA) where YFV epizootics were confirmed just prior collection (Figures 2 and 3).

**Table 2.** Description of YFV-positive mosquito pools. ES: Espírito Santo; RJ: Rio de Janeiro; MG: Minas Gerais.

State	Municipality (Positive pools)	Species	Tested pools (Positive)	MIR^1^	MLE^2^	Relative abund.^3^ (%)	Collection date	Human cases^4^	Days between first YFV signals^5^ and mosq. collections
ES	Domingos_Martins (2)	*Hg. janthinomys Hg. leucocelaenus*	1 (1) 4 (1)	1000 66.6	_ 67.6	3.9 7.1	2/23/17 2/23/17	25	3 (M)
RJ	Macaé (1)	*Hg. janthinomys*	6 (1)	58.8	55.5	17	4/26/17	5	22 (M)
	Maricá (39)	*Hg. janthinomys*	5 (2)	142.8	153.5	0.4	5/5 and 8/5/17	3	18 and 21 (H/M)
		*Hg. leucocelaenus*	100 (35)	81.9	101.6	12.2	5/4-8/17		17-21 (H/M)
		*Ae. scapularis*	72 (1)	2.1	2.1	21.6	5/6/17		19 (H/M)
		*Ae. taeniorhynchus*	164 (1)	0.6	0.6	52.6	5/6/17		20 (H/M)
	Teresópolis (2)	*Hg. janthinomys*	19 (1)	13.1	13.1	18.7	12/19/17	23	5 (M)
		*Hg. leucocelaenus*	18 (1)	19.6	19.1	15.7	12/19/17		5 (M)
	Nova Iguaçú (1)	*Hg. janthinomys*	8 (1)	29.4	29.4	21.6	1/9/18	0	4 (M)
	Valença (15)	*Hg. janthinomys*	40 (12)	70.5	80.1	36.6	1/18,19,24,26/18	40	11, 12, 17, 19 (H)
		*Hg. leucocelaenus*	33 (3)	23.2	23.7	23.6	1/24/18		17 (H)
	Angra dos Reis (Ilha Grande) (1)	*Sa. chloropterus*	1 (1)	1000	_	0.7	2/7/18	57	3 (M)
MG	Belmiro Braga (2)	*Hg. janthinomys*	24 (1)	10.8	10.9	24.5	1/29/18	1	19 (M)
		*Hg. leucocelaenus*	18 (1)	13.5	13.3	23.8	1/18/18		8 (M)
	Juiz de Fora (1)	*Hg. janthinomys*	1 (1)	333.3	_	17.9	1/27/18	43	24 (M)

^1^Minimum Infection Rate = No of positive pools/No of same species adults analyzed × 1000; ^2^Maximum Likelihood Estimate per 1000 mosquitoes = 1− (1−*Y*/*X*)^1/m^ where *Y* is the number of positive pools, *X* is the total number of pools, and *m* is the size of each tested pool; ^3^Relative abundance = (Number of adults of the same species/No total of adults) × 100; ^4^YF human cases detected in the same epidemic period in which mosquito collections were carried out. ^5^Epizootic events in NHPs (M) and/or human (H) hospitalizations with clinical suspicion of YF.

*Haemagogus janthinomys* and/or *Hg. leucocelaenus* were the species found infected in all localities where positive mosquitoes were detected, except in Angra dos Reis – Ilha Grande, RJ, where *Sa. chloropterus* was the only positive species although *Hg. leucocelaenus* was present (Figure 2). *Haemagogus janthinomys* displayed higher infection rates (MIR and MLE) than *Hg. leucocelaenus* in most localities (Table 2). *Aedes scapularis* (MIR = 2.1, MLE = 2.1) and *Ae. taeniorhynchus* (MIR = 0.6, MLE = 0.6) were found infected only once, coincidently in the same municipality (Maricá, RJ), where *Hg. leucocelaenus* (MIR = 81.9, MLE = 101.6) and *Hg. janthinomys* (MIR = 142.8, MLE = 153.5) were also detected naturally infected (Table 2, Figure 3).

Additionally, we tested 976 adult *Hg. leucocelaenus* and 19 *Hg. janthinomys* obtained from eggs collected in the same areas (Domingos Martins, Macaé, Maricá, Valença, Teresópolis and Belmiro Braga) and time in which infected females were detected, but all were negative, providing no evidence of vertical transmission.

Although widespread in municipalities suffering YFV outbreaks, all *Ae. aegypti* specimens were found negative, even when collected around houses inhabited by viremic dwellers infected in the sylvatic cycle.

## Discussion

At the beginning of 2017, YFV reached Brazilian coastal states both in the southeast and northeast (BA), Atlantic Forest regions considered YFV-free and without vaccine recommendation for decades [9,17,20], causing a major sylvatic outbreak and devastating epizootics among NHPs in 2017–2018. During almost 80 years without YFV circulation, the southeast region under influence of the Atlantic Forest underwent significant environmental changes and a remarkable 368% increase in human population density that potentially influenced mosquito fauna distribution, diversity and abundance [11]. Changes in mosquito communities would potentially govern vector species status and might shape arbovirus transmission patterns [21].

Our results were based on extensive mosquito sampling, which covered approximately 1300 km between the northernmost and southernmost surveyed municipalities (Salvador – BA and Paraty – RJ), in the four most affected states, through a combined analysis of mosquito distribution, abundance and YFV natural infections before and during the outbreak. Although there had been no evidence of YFV circulation for nearly 80 years at 28 municipalities where we sampled mosquitoes before the outbreak, traditional YFV vectors were alarmingly detected in most of them (82%), revealing the high local receptivity to sylvatic YFV transmission. Therefore, its establishment in the coastal Atlantic Forest was only a matter of time.

Altogether, results obtained during the outbreak indicate *Hg. janthinomys* and *Hg. leucocelaenus* as the main sylvatic YFV vectors in the region. The genome sequencing of viral RNA detected in these mosquitoes (e.g. GenBank accession numbers MF423373 and MF423374) confirmed the occurrence of a unique molecular signature of fixed amino acid mutations in highly conserved positions at NS3 and NS5 proteins in YFV causing the current Brazilian outbreak [13,16]. Other taxa found naturally infected, such as *Sa. chloropterus*, *Ae. scapularis* and *Ae. taeniorhynchus* appear to have a local or secondary role and, therefore, low epidemiological importance either because of reduced abundance and distribution (*Sa. chloropterus*), or as for the low infection rates (*Ae. scapularis*) combined with distribution limited to coastal lowlands (*Ae. taeniorhynchus*). Noteworthy, natural infections were detected only in mosquitoes captured between 3 and 24 days after glimpsing the first signal of YFV circulation (mostly epizootics) in the respective area, and no vertical transmission in mosquitoes was detected. A total of 5,703 mosquitoes belonging to 84 other species tested negative and showed no obvious role in YFV transmission in this outbreak.

*Haemagogus janthinomys* has been found several times infected with YFV in Brazil and other American countries and is considered the primary vector across Brazilian biomes for the last decades, namely the Amazon endemic region, the emergence zones in the transition between Amazonia and *Cerrado*, as well as in the *Cerrado stricto sensu* [21,22]*.* It had been also recognized as primary vector in the 1930–1940s epidemics in the Atlantic Forest [23]. Our current data reinforce the key role of *Hg. janthinomys* in the 2016–2018 outbreak, that is: density, abundance and distribution increasing during the outbreak (3.0 fold), highest displayed infection rates and favorable behavior, as discussed hereafter. *Haemagogus leucocelaenus*, whose role in sylvatic YFV transmission in the Americas was almost neglected until last decade, is herein considered as primary vector due to its very high distribution and abundance in surveyed municipalities during the outbreak as well as the noteworthy natural infection rates. During the investigation of YFV epidemics in inland southern Brazil, where *Hg. janthinomys* was not found [24,25], *Hg. leucocelaenus* was considered to play an important role in the transmission, although still regarded as secondary vector. The species was also found naturally infected in São Paulo during the sylvatic YF outbreak in 2009, when *Hg. janthinomys* and *Sa. chloropterus* were tested negative [26]. Although, *Hg. janthinomys* had higher infection rate values than *Hg. leucocelaenus* in most surveyed YFV foci as it is usually described in the literature [22], the latter occurred in greater abundance and was more distributed across the southeastern YFV transmission territory, which reinforces its importance in the maintenance and dissemination of YFV in this region. In all municipalities where YFV was detected in mosquitoes, *Hg. janthinomys* or both *Hg. janthinomys* and *Hg. leucocelaenus* (56%) were found infected, except for Ilha Grande where only *Sa. chloropterus* was found carrying the virus. Curiously, natural infections of both species in the same transmission area was described in one RJ site during the 1930s [27]. This suggests that the concurrence of the two species in YFV transmission may be typical and recurrent in the coastal Atlantic Forest, distinctly from other YFV endemic or epidemic South American biomes like the Amazon and *Cerrado*.

Species of *Haemagogus* exhibit primatophilic habits [28], which facilitates their contact with YFV infected NHPs and virus transmission to both human and NHPs. Collectively, their competence to amplify, disseminate and transmit the virus [28–30] as well as the high abundance, distribution, primatophilic behavior, arboreal feeding habits and large flight range may have contributed to the magnitude, severity and rapidity of spread of the 2016–2018 outbreak in this region [31,32]. *Haemagogus leucocelaenus* seems to exhibit greater plasticity of habitats and blood feeding patterns than *Hg. janthinomys* [24,33]. *Haemagogus leucocelaenus* may colonize secondary and modified forest patches, while *Hg. janthinomys* would be more stringent in terms of climatic and environmental conditions [24,34]. While *Hg. janthinomys* bites much more frequently at the canopy level, *Hg. leucocelaenus* usually attacks on lower forest strata, although both mosquitoes may disperse vertically in the forest [30,31]. Indeed, we captured an impressive number of *Hg. janthinomys* (4th most abundant species during the outbreak) at the ground level in the forest, as well as in the forest fringe and in open fields. The lower mean tree canopy height and vegetation density in most surveyed municipalities may sustain less amount and diversity of vertebrate hosts, which may have forced *Hg. janthinomys* to explore the ground level and the open fields. In addition, the topography of forest fragments in most YFV affected municipalities in the southeast may further influence this behavior, as previously suggested to occur with the canopy feeder mosquito *Anopheles cruzii* in the region [35,36]. The combination of behavioral and biological characteristics of these two *Haemagogus* species may also help to understand the estimates of virus spread of 3.5–5 km/day in this Brazilian outbreak [11,13,37]. In fact, *Hg. janthinomys* and *Hg. leucocelaenus* have high dispersion capacity between forest fragments, and may bite distant from the woods and even indoors [11,37,38]. It is important to mention that we found infected *Hg. janthinomys* in forest fragments as small as 7 hectares, some of which are contiguous to urban neighborhoods and remote urbanized high income borough settled in recently cut forest valleys. The growth of cities and the search for a more bucolic life have put people closer to fragments of forest increasing the exposure of people to the sylvatic mosquito bites in this region [11]. Therefore, taking into account the estimated flight range of these main vectors [37], vaccination campaigns in affected municipalities must consider not only people visiting or living in the close vicinity of forest, but also those who live or circulate at distances as large as 12 km from early detected epizooty.

This is the first time that *Sa. chloropterus* is found infected with YFV in the Atlantic Forest biome. The infected *Sa. chloropterus* was recovered when the outbreak reached a large island around 2 km apart from the continent (Ilha Grande, in Angra dos Reis) where we did not find any *Hg. janthinomys,* both before and during the outbreak. At the time of mosquito collection, we found 10 carcasses and 5 dying howler-monkeys on the inspected trails. Interestingly we had found *Hg. leucocelaenus* in collections made before the outbreak. *Sabethes chloropterus* showed low abundance and was limited in distribution throughout the sampled area and time, a characteristic previously described [39,40]. Its canopy-feeding behavior and essentially sylvatic distribution may facilitate the contact with infected NHPs. But, our findings suggest a secondary role of *Sa. chloropterus* as YFV vector in this region of the country.

In contrast to the above-mentioned *Haemagogus* and *Sabethes* mosquitoes*, Aedes scapularis* and *Ae. taeniorhynchus* are opportunistic mosquitoes, whose biting peak occurs primarily in twilight at ground level of fragmented forest, the forest edge and open fields [41]. Thus, the opportunities of these *Aedes* mosquitoes to bite NHPs in the forest canopy are limited. *Aedes scapularis* is experimentally competent to transmit YFV [42,43], and occasionally becomes infected in nature [21]. Nevertheless, its role in the current outbreak, even as a secondary vector, seems to be little relevant as we found only one positive pool despite this species’ great abundance and distribution across YFV foci, both in the lowlands and mountain slopes. We believe our report of YFV natural infection in *Ae. taeniorhynchus* is novel. However, as we examined whole bodies of non-blood-fed mosquitoes, we cannot ensure whether YFV replication would be only limited to the midgut or already present in the salivary glands of the infected specimens. While YFV outbreak affected sites of various reliefs including mostly mountain valleys and slopes, the distribution of *Ae. taeniorhynchus* is limited to the coastal lowlands. Its competence to experimentally transmit YFV is controversial to null [43,44]. Our detections of natural infections in these *Aedes* species occurred exclusively in a forest fragment undergoing a sylvatic transmission of great force illustrated by records of very recently confirmed epizootics in howler-monkeys and human fatal cases nearby and where we collected 40 positive pools of *Hg. janhtinomys* and *Hg. leucocelaenus* in one week*.* So, it is likely that *Ae. scapularis* and *Ae. taeniorhynchus* only become infected in environments and moments when the availability of viremic hosts infected by the bite of *Haemagogus* mosquitoes is very high. Thus, natural infections in these mosquitoes, especially in *Ae. taeniorhynchus,* should be considered with caution as they do not assure this species playing any important role in the spread of YFV.

Despite our large sample effort, species considered as potential vectors and found naturally infected with YFV in other areas (e.g. *Ae. serratus* [24], *Ae. albopictus* [43], *Sa. albiprivus* and *Psorophora* species [29,45–48]) were negative in the present study, even when captured in large quantities and in sympatry with the infected *Haemagogus*. *Sa. albiprivus* was the most abundant and well distributed species of *Sabethes*, both in arid areas of *Cerrado*, in dense Atlantic Forest as well as in transition between these biomes, and has proved to be competent to experimentally transmit YFV [29]. Therefore, *Sa. albiprivus* may play a very secondary role on YFV maintenance, even if not detected in the present study.

We did not find any natural infection when analyzing numerous *Ae. albopictus* from areas with large numbers of human cases and/or confirmed epizootics (e.g. Ilha Grande, Valença and Juiz de Fora). Brazilian *Ae. albopictus* may experimentally transmit YFV of the South American genotype, and it has been shown that YFV has the potential for adaptation to this mosquito with augmentation of virus titers in the saliva following successive contacts [29,49–51]. Additionally, this species was the most disseminated in the municipalities sampled during the outbreak in the southeast, the Brazilian region most infested by this mosquito [52]. Several authors warn that *Ae. albopictus* may act as bridge vector and would represent a threat of YFV reurbanization or facilitating enzootic spillovers with establishment of YFV into an intermediate/rural cycle due to its ecological plasticity and ubiquitous environmental distribution [11,52,53]. An important overlap of expanded niches of the sylvatic primary vectors, *Hg. janthinomys* and *Hg. leucocelaenus,* and the anthropic ones, like *Ae. albopictus,* in the Atlantic Forest biome has been observed in the last decades [11]. Therefore, it is advised to urgently design and apply surveillance and control measures concerning this mosquito in areas with transmissions and at risk.

Urban YF has not been recorded in Brazil since 1942. However, there is a great concern about urban YFV reemergence due to high infestation indices of *Ae. aegypti* in periurban and urban areas very close to the sylvatic cycles in the low vaccination coverage municipalities, such as those affected by the outbreak in southeastern Brazil [12,15,20]. All *Ae. aegypti* specimens we captured during the outbreak, including those sampled around houses inhabited by viremic humans infected in the sylvatic cycle, tested negative for YFV. However, the low vaccination coverage, the presence of *Hg. leucocelaenus* in several urban parks, the proximity of NHPs in several cities with the arrival of YFV-viremic humans seeking medical care in urban centers infested with *Ae. aegypti* are among the factors that may increase the risks of YFV reurbanization in the south and southeast, the most populated region of the country [11]. Most surveyed municipalities in southeastern Brazil have frequently endured urban epidemics of other *Ae. aegypti-*transmitted viruses, including dengue, chikungunya and Zika. It fact, YFV circulated intensely in 2017–2018 where *Ae. aegypti* is very active. Together, these facts indicate that reducing *Ae. aegypti* populations and vaccinating urban populations near sylvatic outbreaks is more critical than ever.

The 2016–2018 sylvatic outbreak was the most severe in the last eight decades. Understanding the causes of this severity needs virological, primatological, ecological, epidemiological and immunological studies [11,13,16,54]. YFV transmission is a complex and multifactorial phenomenon involving social, ecological and biological issues, among which the entomological component is crucial. Here, by describing the distribution and abundance of potential transmitters and defining the primary vectors throughout the region touched by the outbreak, we could advise proper control measures as well as assemble essential knowledge on this intricate epidemiological event.

Entomological and virological surveillance must be urgently and permanently considered from northeast to south Brazil to rapidly define receptive and vulnerable areas as well as early detection of virus circulation, for better assessment of the risk areas and prediction of future spread, and thus target a quick extension of vaccination in expanded risk areas and prioritize the most affected age group using mobile immunization units, simultaneously moving toward the universal routine YFV vaccination for the entire Brazilian population.

## Material and methods

*Study chronology*: Mosquitoes collections were performed in 44 municipalities in two distinct epidemiological situations: before and during the YFV outbreak. From May/2015 to June/2017, 12–15 days mosquito samplings were carried out in 28 municipalities before any local identification of YFV transmission, in order to evaluate receptivity for YF reemergence and early detect this arbovirus circulation. From Jan/2017 to May/2018, 1–8 days mosquito collections were conducted in 21 municipalities with suspected or confirmed YFV foci, i.e. where human cases or epizootic events had just been locally recorded. Among these 21 municipalities, 16 were surveyed for the first time and 5 had already been sampled before the outbreak (Figure 1).

*Study areas:* The criteria for selection of sampling municipalities were distinct according to the above-mentioned aims and epidemiological situations, i.e. before or during local YFV circulation (Figure 1). In the first situation, surveyed municipalities (*N* = 28) belonged to RJ and to bordering states (MG and SP). These municipalities were selected to include a variety of ecological and environmental conditions.

Twenty-one municipalities with YFV transmission locally confirmed were surveyed, being 20 in the three most affected states (MG, ES and RJ), that recorded a total of 73.5% of confirmed human cases, and one in Bahia (BA) where only NPH infections were confirmed. We selected five out these municipalities because they had been surveyed prior to local YFV transmission, and the remaining 16 municipalities were chosen taking into account the local incidence of human cases and epizootic records and the proximity to the great metropolitan areas in RJ, ES, MG and BA (Figure 1).

*Entomological surveys*: In all 44 surveyed municipalities (Figure 1), adult mosquitoes were caught with aspirators and nets during incursions into forest patches and their edges as well as near houses. Specimens were frozen in liquid N_2_ or dry ice in the field, and kept under the same conditions of freezing during transport to our laboratory at Instituto Oswaldo Cruz (IOC) in Rio de Janeiro.

In seven municipalities (Belo Horizonte and Simonésia – MG, Domingos Martins and Serra – ES, Maricá and Casimiro de Abreu – RJ, and Salvador – BA), adult mosquitoes were also collected using BG-Sentinel traps (Biogentes) baited with CO_2._ Twelve BG traps were continuously operated per area for 3–5 days. They were installed at each 100  m (0, 100, 200, and 300  m) along three transects from the edge to deep into the forest fragments, as described in more details elsewhere [53].

Ovitraps baited with an infusion of dry leaves found on forest ground and three wooden paddles each were settled for 7–12 days at the canopy of 15–30 trees per area. Paddles found with eggs were immerged in dechlorinated water for egg hatching, and larvae were reared until adult stage in the laboratory. Larvae were also sampled from all detected natural larval habitats (e.g. bromeliads, trees-holes, bamboo internodes) and reared until adult.

In the laboratory, field and laboratory emerged adult mosquitoes were identified under a stereomicroscopy on a cold table [55]. Voucher specimens were deposited at the CCuli Collection, at IOC (url: http://cculi.fiocruz.br/index). *Haemagogus janthinomys*, a common species widely distributed across South America, and *Haemagogus capricornii* are sympatric in southeast Brazil. Their adult females and immature forms are morphologically indistinguishable, the few distinctive characters are in male genitalia [56]. When available, male genitalia of *Haemagogus* collected in all municipalities were examined. However, males with the phenotype corresponding to *Hg. capricornii* were found only in Valença (RJ). Therefore, we used the name *janthinomys* to refer to specimens potentially belonging to either taxa.

The major part of captured adults was pooled (≤10 individuals each) according to species, sampling locality and date, and subsequently homogenized in 250–1000 µL of L-15 culture medium by using the Precellys 24^®^ tissue homogenizer in bead tubes. Homogenates were kept at −80°C for posterior viral genome detection. Only non-blood-fed mosquitoes were analyzed.

*Virus detection*: After centrifugation (9600 *g*, 10  min, 4°C), RNA was extracted from 140 µL of supernatant using the Qiagen RNA Viral Kit following the manufacturer's recommendations. RNA from mosquitoes obtained in the first half 2017 were screened by conventional RT-PCR while the remaining samples were examined by RT-qPCR. Details for RT-PCR protocol were previously described [16]. Briefly, the set of primers utilized in the conventional PCR were 5′-CTGTGTGCTAATTGAGGTGCATTG-3′ and 5′-ATGTCATCAGGCTCTTCTCT-3′, targeting nucleotides 9 to 663, between 5′ and PrM regions of the YFV genome. Infections were diagnosed by the specific detection of this single amplicon with the likely YFV amplicon size of 650 bp. Obtained amplicons were purified using Qiagen QIAquik PCR purification kit following the manufacturer’s recommendation. For confirmation, the amplicons were directly sequenced without molecular cloning. Nucleotide sequencing reactions were performed using the ABI BigDye terminator V3.1 Ready Reaction Cycle Sequencing Mixture (Applied Biosystems) according to manufacturer’s recommendations. Nucleotide sequence was determined by capillary electrophoresis at the sequencing facility of Fiocruz-RJ (RPT01A – Sequenciamento de DNA – RJ). Raw sequence data were aligned and edited using the SeqMan module of LaserGene (DNASTAR Inc.). Edited nucleic acid sequences were compared to other YFV strains available at the Gen-Bank database using The Basic Local Alignment Search Tool (BLAST) (url: http://blast.ncbi.nlm.nih.gov/Blast.cgi).

For RT-qPCR, viral RNA was reverse transcribed and amplified using the TaqMan Fast Virus 1-Step Master Mix (Applied Biosystems) in an Applied Biosystems StepOnePlus Instrument. For each reaction we used 300 nM forward primer (5′-GCTAATTGAGGTGCATTGGTCTGC-3′, genome position 15–38), 600 nM reverse primer (5′-CTGCTAATCGCTCAACGAACG-3′, genome position 83–103) and 250 nM probe (5′FAM-ATCGAGTTGCTAGGCAATAAACAC-3′TAMRA, genome position 41–64). Samples were run in duplicate, with deionized water serving as negative control for extraction and PCR reactions. The reverse transcription was performed at 50°C for 5  min. The qPCR conditions were 95°C for 20 s, followed by 40 amplification cycles of 95°C for 15 s and 60°C for 1  min. Copy numbers of YF genomic RNA were calculated by absolute quantitation using a standard curve for each run. To construct the standard curve, an amplicon was cloned comprising the genomic region 1 to 865 of the isolate ES-504 (GeneBank accession number: KY885000) using pGEM-T Easy Vector (Promega) to serve as a template for *in vitro* transcription. The RNA was transcript with mMessage mMachine High Yield Capped RNA Transcription Kit (Invitrogen) using SP6 enzyme and purified using MEGAclear Kit (Ambion) according to manufacturer’s instructions. The purity of the transcript was verified using NanoDrop 8000 Spectrophotometer (Thermo Scientific), and the concentration of the RNA was determined using Qubit 2.0 Fluorometer (Invitrogen). The standard curve was generated by serial ten-fold dilution (ranging from 10 to 10^9^ copies/reaction) of the transcript. The limit of detection under standard assay conditions was approximately 40 viral RNA copies/mL. Confirmation of YFV diagnose was done by amplifying and sequencing the 650 bp amplicon as described above.

Standardization of RT-qPCR assays was done from serial dilutions (10^−1^ to 10^−10^) of one confirmed wild mosquito positive sample, mixed or not with homogenates of negative *Ae. aegypti* from our laboratory colony. The sensitivity of the test was compared in triplicate with that of conventional RT-PCR. Conventional RT-PCR and RT-qPCR detected the virus genome until 10^−7^ and 10^−9^ dilution, respectively.

*Data analyses*: Quantitative and qualitative fauna data were analyzed in Microsoft Excel software. Two infections rates were used: Minimum Infection Rate (MIR) was calculated by dividing the number of infected pools by the total number of adult mosquitoes of the same species collected in the same area, multiplied per 1000. Maximium Likelihood Estimate (MLE) per 1000 adult mosquitoes was obtained using the formula 1− (1−*Y*/*X*)^1/*m*^ where *Y* is the number of positive pools, *X* is the total number of pools, and m is the size of each tested pool [57]. Maps were constructed in Arcgis webmap version.

*Ethical statements*: Mosquito collections in the Atlantic Forest were approved by local environmental authorities (SISBIO-MMA licenses 54707–2 and 52472–2, INEA license 012/2016012/2016 and PNMNI license 001/14–15). This study did not involve endangered or protected species.

## Supplementary Material

Supplemental Material
