# Supplementary material for: Haemagogus leucocelaenus and Haemagogus janthinomys are the primary vectors in the major yellow fever outbreak in Brazil, 2016–2018
Source: Emerg Microbes Infect. 2019 Feb 1;8(1):218–31. doi: 10.1080/22221751.2019.1568180 (PMC6455131; doi:10.1080/22221751.2019.1568180)
Supplement: Supplemental Material [file TEMI_A_1568180_SM4764.zip › Table_Suppl_1_RLO.docx]

Supplementary table 1: Density of mosquito species^1^ captured before the YFV outbreak in each municipality. Dates of collections are in parenthesis: AP: Além Paraíba (May 2016), AR: Angra dos Reis (Jun 2017), BH: Belo Horizonte (Feb 2017), CM: Carmo (May 2016) , CO: Cordeiros (Jan 2017), GP: Guapimirim (Dec 2016), IM: Itamonte (Nov 2016), IT: Itatiaia (Nov 2016), MC: Macaé (May and Aug 2015), MG: Magé (Dec 2016), MP: Miguel Pereira (Oct 2015 and Mar 2016), NF: Nova Friburgo (Mar 2016), PA: Paraty (Jun 2017), PT: Petrópolis (Oct 2015), PR: Piraí (Apr 2017) PO: Porciúncula (Jan 2017), QL: Queluz (Nov 2016), RE: Resende (Jul 2017), RC: Rio Claro (May 2017), RJ: Rio de Janeiro (Apr-May 2017), SS: São Sebastião do Alto (Mar 2017), SQ: Saquarema (Mar 2017), SJ: Silva Jardim (Mar 2017), SU: Sumidouro (Dec 2016), TR: Teresópolis (Dec 2016), TS: Três Rios (May 2016).

|  | **AP** | **AR** | **BH** | **CM** | **CO** | **GP** | **IM** | **IT** | **MC** | **MG** | **MP** | **NF** | **PA** | **PT** | **PR** | **PO** | **QL** | **RE** | **RC** | **RJ** | **SS** | **SQ** | **SJ** | **SU** | **TR** | **TS** | **TOTAL** |
| --- | --- | --- | --- | --- | --- | --- | --- | --- | --- | --- | --- | --- | --- | --- | --- | --- | --- | --- | --- | --- | --- | --- | --- | --- | --- | --- | --- |
| **Human cases** | 0 | 0 | 0 | 0 | 0 | 0 | 0 | 0 | 0 | 0 | 0 | 0 | 0 | 0 | 0 | 0 | 0 | 0 | 0 | 0 | 0 | 0 | 0 | 0 | 0 | 0 | **_** |
| **Positive pools** | 0 | 0 | 0 | 0 | 0 | 0 | 0 | 0 | 0 | 0 | 0 | 0 | 0 | 0 | 0 | 0 | 0 | 0 | 0 | 0 | 0 | 0 | 0 | 0 | 0 | 0 | **_** |
| *Ae. aegypti* | _ | _ | 6.9 | _ | _ | _ | _ | _ | _ | _ | _ | _ | _ | _ | 2.5 | _ | _ | _ | _ | 33.3 | _ | _ | _ | _ | _ | _ | **42.7** |
| *Ae. albopictus* | _ | 6.7 | 8.8 | 130.0 | 50.0 | 0.8 | _ | _ | 2.4 | 22.5 | _ | _ | _ | _ | 75.0 | 120.0 | _ | _ | 2.5 | 155.0 | 25.0 | 15.0 | 36.3 | _ | _ | 40.0 | **689.9** |
| *Ae. fluviatilis* | _ | 5.0 | _ | _ | _ | _ | _ | _ | _ | _ | _ | _ | _ | _ | _ | _ | _ | _ | _ | _ | _ | _ | _ | _ | 1.0 | _ | **6.0** |
| *Ae. fulvithorax* | _ | _ | 0.6 | _ | _ | 1.7 | _ | _ | _ | _ | _ | _ | 2.5 | _ | _ | _ | _ | _ | _ | _ | _ | _ | _ | _ | _ | _ | **4.8** |
| *Ae. scapularis* | _ | 6.7 | 347.5 | _ | 50.0 | 8.3 | 15.0 | 1.0 | 40.0 | _ | 1.2 | 37.5 | 35.0 | _ | 10.0 | 120.0 | 5.0 | _ | 80.0 | 10.8 | _ | 235.0 | 276.3 | 10.0 | 2.0 | _ | **1291.2** |
| *Ae. serratus* | _ | _ | _ | _ | 20.0 | _ | _ | _ | 0.9 | _ | _ | _ | 20.0 | _ | _ | 50.0 | _ | _ | 2.5 | _ | _ | _ | 60.0 | _ | _ | _ | **153.4** |
| *Ae. taeniorhynchus* | _ | _ | _ | _ | _ | _ | _ | _ | _ | _ | _ | _ | _ | _ | 35.0 | _ | _ | _ | _ | _ | _ | 1025.0 | _ | _ | _ | _ | **1060.0** |
| *Ae. terrens* | _ | _ | _ | _ | _ | 15.0 | _ | 1.0 | 0.3 | 5.0 | 14.6 | 10.0 | _ | 15.0 | _ | _ | _ | _ | _ | _ | _ | _ | _ | 10.0 | 5.0 | _ | **75.9** |
| *Aedeomyia sp.* | _ | _ | _ | _ | _ | _ | _ | _ | _ | _ | _ | _ | _ | _ | _ | _ | _ | _ | 10.0 | _ | _ | _ | _ | _ | _ | _ | **10.0** |
| *Aedes sp.* | _ | _ | 0.6 | _ | _ | _ | _ | _ | _ | _ | _ | _ | _ | _ | _ | _ | _ | _ | _ | _ | 52.5 | _ | _ | _ | _ | _ | **53.1** |
| *An. bellator* | _ | _ | _ | _ | _ | _ | _ | _ | _ | _ | 0.8 | _ | _ | _ | _ | _ | _ | _ | _ | _ | _ | _ | _ | _ | _ | _ | **0.8** |
| *An. cruzii* | _ | _ | _ | _ | _ | _ | _ | _ | _ | _ | 158.8 | 3.8 | 2.5 | 465.0 | _ | _ | _ | _ | _ | _ | _ | _ | _ | _ | 1.0 | _ | **631.1** |
| *An. fluminensis* | _ | _ | _ | _ | _ | _ | _ | _ | _ | _ | _ | _ | _ | _ | _ | _ | _ | _ | _ | _ | _ | _ | 5.0 | _ | _ | _ | **5.0** |
| *An. lutzi* | _ | _ | _ | _ | _ | _ | _ | _ | _ | _ | 0.4 | _ | _ | _ | _ | _ | _ | _ | _ | _ | _ | _ | _ | _ | _ | _ | **0.4** |
| *An. mediopunctatus* | _ | 1.7 | _ | _ | _ | _ | _ | _ | _ | _ | _ | _ | _ | _ | _ | _ | _ | _ | _ | _ | _ | _ | _ | _ | _ | _ | **1.7** |
| *An. neivai* | _ | _ | _ | _ | _ | _ | _ | _ | _ | _ | 0.4 | _ | _ | _ | _ | _ | _ | _ | _ | _ | _ | _ | _ | _ | _ | _ | **0.4** |
| *Anopheles sp.* | _ | _ | _ | _ | _ | 0.8 | 17.5 | _ | _ | _ | 7.3 | 3.8 | _ | _ | _ | _ | _ | _ | _ | _ | _ | _ | _ | _ | _ | _ | **29.4** |
| *Cq. venezuelensis* | _ | _ | _ | _ | _ | _ | _ | _ | _ | _ | _ | _ | _ | _ | 2.5 | _ | _ | _ | _ | _ | _ | _ | 2.5 | _ | _ | _ | **5.0** |
| *Culex coronator'* | _ | _ | 0.6 | _ | _ | _ | _ | _ | _ | _ | _ | _ | _ | _ | _ | _ | _ | _ | _ | _ | _ | _ | _ | _ | _ | _ | **0.6** |
| *Culex sp.* | _ | 3.3 | 3.1 | 10.0 | _ | 5.8 | 7.5 | _ | 6.1 | _ | 6.2 | 23.8 | _ | _ | 5.0 | _ | _ | 2.5 | 2.5 | 3.3 | _ | _ | _ | _ | 1.0 | _ | **80.1** |
| *Culicidae* | _ | _ | _ | _ | _ | 0.8 | _ | _ | _ | _ | 1.9 | _ | _ | _ | _ | _ | _ | _ | _ | _ | _ | _ | _ | _ | _ | _ | **2.8** |
| *Cx. nigripalpus* | _ | 1.7 | _ | 40.0 | _ | 8.3 | _ | _ | _ | _ | _ | _ | _ | _ | _ | _ | _ | _ | _ | 4.2 | _ | _ | 1.3 | _ | _ | _ | **55.4** |
| *Cx. quinquefasciatus* | _ | _ | 2.5 | _ | _ | _ | _ | _ | _ | _ | _ | _ | _ | _ | 12.5 | _ | _ | _ | _ | _ | _ | _ | _ | _ | _ | _ | **15.0** |
| *Haemagogus sp.* | _ | _ | _ | _ | _ | _ | _ | _ | 0.3 | _ | _ | _ | _ | _ | _ | _ | _ | _ | _ | _ | _ | _ | _ | _ | _ | _ | **0.3** |
| *Hg. janthinomys* | _ | _ | 0.6 | 30.0 | 30.0 | 45.0 | _ | 1.0 | _ | 27.5 | 0.8 | 7.5 | _ | _ | _ | 10.0 | 6.7 | _ | _ | _ | _ | _ | _ | 30.0 | _ | _ | **189.1** |
| *Hg. leucocelaenus* | _ | 6.7 | 21.9 | 60.0 | 10.0 | 60.8 | _ | 5.0 | 7.3 | _ | 2.3 | 7.5 | 5.0 | _ | 7.5 | 10.0 | 8.3 | 2.5 | _ | 282.5 | 2.5 | _ | 6.3 | 10.0 | 7.0 | _ | **523.0** |
| *Li. durhamii* | _ | 1.7 | 8.1 | 40.0 | 20.0 | 1.7 | _ | 30.0 | 1.5 | _ | 2.3 | 35.0 | 10.0 | _ | _ | 10.0 | 1.7 | 2.5 | 5.0 | 7.5 | _ | _ | 1.3 | _ | 21.0 | _ | **199.2** |
| *Li. pseudomethisticus* | _ | _ | _ | 10.0 | _ | 0.8 | _ | 42.0 | _ | _ | 0.4 | 15.0 | _ | _ | _ | _ | _ | 5.0 | _ | _ | _ | _ | _ | _ | _ | _ | **73.2** |
| *Limatus sp.* | _ | _ | _ | _ | _ | _ | _ | _ | _ | _ | _ | _ | _ | _ | _ | _ | _ | _ | _ | _ | 2.5 | _ | _ | _ | _ | _ | **2.5** |
| *Ma. indubitans* | _ | _ | _ | _ | _ | _ | _ | _ | _ | _ | _ | _ | _ | _ | _ | _ | _ | _ | _ | _ | _ | _ | 1.3 | _ | _ | _ | **1.3** |
| *Ma. titillans* | _ | _ | _ | _ | _ | _ | _ | _ | _ | _ | _ | _ | _ | _ | 20.0 | _ | _ | _ | _ | _ | _ | _ | 1.3 | _ | _ | _ | **21.3** |
| *Mansonia sp.* | _ | _ | _ | _ | 20.0 | _ | _ | _ | 1.8 | _ | _ | _ | _ | _ | _ | 20.0 | _ | _ | _ | _ | _ | _ | _ | _ | _ | _ | **41.8** |
| *On. personatum* | _ | _ | _ | _ | _ | 15.0 | _ | 1.0 | _ | _ | 9.6 | 23.8 | _ | _ | _ | _ | 3.3 | _ | _ | _ | _ | _ | _ | _ | 14.0 | _ | **66.7** |
| *Ps. albipes* | _ | _ | _ | _ | _ | _ | _ | _ | _ | _ | _ | _ | _ | _ | _ | _ | _ | _ | _ | _ | _ | _ | 115.0 | _ | _ | _ | **115.0** |
| *Ps. ferox* | _ | _ | 10.0 | 50.0 | 200.0 | 1.7 | _ | _ | 9.7 | _ | _ | _ | 12.5 | _ | 42.5 | 20.0 | 1.7 | _ | 97.5 | 0.8 | _ | _ | 50.0 | _ | _ | _ | **496.4** |
| *Ps. lutzii/amazonica* | _ | _ | _ | _ | _ | _ | _ | _ | _ | _ | _ | _ | 5.0 | _ | _ | _ | _ | _ | _ | _ | _ | _ | _ | _ | _ | _ | **5.0** |
| *Ps. pseudomelanota'* | _ | _ | _ | _ | _ | _ | _ | _ | _ | _ | _ | _ | _ | _ | _ | _ | _ | _ | _ | _ | _ | _ | 1.3 | _ | _ | _ | **1.3** |
| *Psorophora sp.* | _ | _ | 1.9 | _ | _ | _ | _ | _ | 2.4 | _ | _ | _ | _ | _ | _ | 30.0 | _ | _ | 5.0 | _ | _ | _ | _ | _ | _ | _ | **39.3** |
| *Ru. cerqueirai* | _ | _ | _ | _ | _ | 10.8 | _ | _ | _ | _ | _ | 1.3 | _ | _ | _ | _ | _ | _ | _ | _ | _ | _ | _ | _ | 2.0 | _ | **14.1** |
| *Ru. frontosa* | _ | 5.0 | _ | 20.0 | _ | 15.8 | _ | _ | _ | 2.5 | 1.2 | 27.5 | 7.5 | 35.0 | _ | _ | 11.7 | 2.5 | _ | _ | _ | _ | _ | _ | 2.0 | _ | **130.7** |
| *Ru. humboldti* | _ | 5.0 | _ | _ | _ | 17.5 | _ | _ | 0.6 | _ | 6.2 | 31.3 | _ | 55.0 | _ | _ | _ | _ | _ | _ | _ | _ | _ | _ | 20.0 | _ | **135.5** |
| *Ru. reversa/theobaldi* | _ | _ | _ | 70.0 | _ | 0.8 | _ | _ | 0.3 | _ | _ | _ | _ | _ | _ | _ | _ | _ | _ | _ | _ | 5.0 | _ | _ | _ | _ | **76.1** |
| *Runchomyia sp.* | _ | 1.7 | _ | 10.0 | _ | 11.7 | 2.5 | 1.0 | _ | _ | 11.2 | 27.5 | _ | _ | _ | _ | _ | _ | _ | _ | _ | _ | _ | _ | 2.0 | _ | **67.5** |
| *Sa. albiprivus* | _ | _ | 76.9 | _ | _ | 0.8 | _ | 1.0 | 0.9 | _ | _ | _ | _ | _ | _ | _ | _ | _ | _ | _ | 2.5 | _ | _ | 30.0 | _ | _ | **112.1** |
| *Sa. aurescens* | _ | 1.7 | _ | _ | _ | 10.0 | _ | 3.0 | _ | _ | 1.9 | 5.0 | _ | 10.0 | _ | _ | _ | _ | _ | _ | _ | _ | _ | 20.0 | 2.0 | _ | **53.6** |
| *Sa. chloropterus* | _ | _ | _ | _ | _ | 3.3 | _ | _ | _ | 2.5 | 1.9 | _ | 2.5 | _ | _ | _ | _ | _ | _ | _ | _ | _ | _ | _ | _ | _ | **10.3** |
| *Sa. fabricii/undosus* | _ | _ | _ | _ | _ | _ | _ | _ | _ | _ | 1.2 | _ | _ | 15.0 | _ | _ | _ | 7.5 | _ | _ | _ | _ | _ | _ | _ | _ | **23.7** |
| *Sa. identicus* | 7.5 | _ | _ | _ | _ | 0.8 | _ | _ | 0.3 | _ | _ | _ | _ | _ | _ | _ | _ | _ | _ | _ | _ | _ | 1.3 | _ | _ | _ | **9.9** |
| *Sa. intermedius* | _ | _ | _ | _ | _ | 0.8 | _ | 3.0 | _ | 2.5 | _ | _ | _ | _ | _ | _ | _ | 7.5 | _ | _ | _ | _ | _ | _ | _ | _ | **13.8** |
| *Sa. melanonymphe* | 5.0 | _ | _ | _ | _ | _ | _ | _ | _ | _ | 1.9 | 2.5 | _ | _ | _ | _ | 1.7 | _ | _ | _ | _ | _ | _ | _ | 7.0 | _ | **18.1** |
| *Sa. purpureus'* | _ | _ | _ | _ | _ | _ | _ | _ | _ | _ | _ | _ | _ | _ | _ | _ | _ | _ | _ | _ | _ | _ | _ | 10.0 | _ | _ | **10.0** |
| *Sa. soperi* | _ | _ | _ | _ | _ | _ | _ | _ | _ | _ | _ | _ | _ | _ | _ | _ | _ | 2.5 | _ | _ | _ | _ | 1.3 | _ | _ | _ | **3.8** |
| *Sa. xyphydes* | _ | _ | _ | _ | _ | _ | _ | _ | 0.6 | _ | _ | _ | _ | _ | _ | _ | _ | _ | _ | _ | _ | _ | _ | _ | _ | _ | **0.6** |
| *Sabethes sp.* | _ | _ | 10.0 | _ | _ | 5.0 | _ | _ | 2.1 | 2.5 | 15.4 | 6.3 | _ | 15.0 | _ | 30.0 | _ | _ | _ | _ | 2.5 | _ | _ | _ | 1.0 | _ | **89.8** |
| *Sh. fluviatilis* | _ | _ | _ | _ | _ | 0.8 | 2.5 | 1.0 | _ | _ | 49.6 | 36.3 | _ | 5.0 | _ | _ | 1.7 | 2.5 | _ | _ | _ | _ | _ | 30.0 | 4.0 | _ | **133.4** |
| *Shannoniana sp.* | _ | _ | _ | _ | _ | _ | _ | _ | _ | _ | 10.8 | _ | _ | _ | _ | _ | _ | _ | _ | _ | _ | _ | _ | _ | _ | _ | **10.8** |
| *Tr. castroi/similis* | _ | 18.3 | _ | _ | _ | _ | 7.5 | 5.0 | _ | _ | _ | _ | _ | _ | _ | _ | 1.7 | _ | _ | _ | _ | _ | _ | _ | _ | _ | **32.5** |
| *Tr. compressum* | _ | 1.7 | _ | _ | _ | _ | _ | _ | _ | _ | _ | _ | _ | _ | _ | _ | _ | 2.5 | _ | _ | _ | _ | _ | _ | _ | _ | **4.2** |
| *Tr. digitatum* | _ | _ | _ | _ | _ | 15.0 | _ | 6.0 | _ | 2.5 | 2.3 | 3.8 | _ | _ | _ | _ | _ | 2.5 | _ | _ | _ | _ | _ | _ | 1.0 | _ | **33.1** |
| *Tr. pallidiventer* | _ | 10.0 | _ | 20.0 | _ | _ | 12.5 | 2.0 | 0.3 | 5.0 | 6.9 | 15.0 | _ | 15.0 | _ | _ | 6.7 | _ | 2.5 | _ | _ | _ | _ | _ | 1.0 | _ | **96.9** |
| *Trichoprosopon sp.* | _ | _ | _ | _ | _ | 0.8 | _ | _ | _ | _ | _ | _ | _ | _ | 2.5 | _ | _ | _ | _ | _ | _ | _ | _ | _ | _ | _ | **3.3** |
| *Wy. antunesi* | _ | _ | _ | _ | _ | _ | 7.5 | _ | _ | _ | _ | _ | _ | _ | _ | _ | _ | _ | _ | _ | _ | _ | _ | _ | _ | _ | **7.5** |
| *Wy. aporonoma/staminifera* | _ | 8.3 | _ | _ | _ | 3.3 | _ | 1.0 | 0.3 | 2.5 | 0.4 | 5.0 | 7.5 | _ | 7.5 | _ | _ | _ | _ | _ | _ | _ | 3.8 | _ | _ | _ | **39.6** |
| *Wy. bonnei/deanei* | _ | _ | _ | 130.0 | _ | _ | _ | _ | _ | _ | 0.4 | 5.0 | _ | _ | _ | _ | _ | _ | _ | _ | _ | _ | _ | _ | _ | _ | **135.4** |
| *Wy. bourrouli/ forcipenis* | _ | 3.3 | _ | 10.0 | _ | _ | _ | _ | _ | _ | _ | _ | _ | _ | _ | _ | _ | _ | _ | _ | _ | _ | _ | _ | _ | _ | **13.3** |
| *Wy. cerqueirai* | _ | _ | _ | _ | _ | _ | _ | _ | _ | _ | _ | 1.3 | _ | _ | _ | _ | _ | _ | _ | _ | _ | _ | _ | _ | _ | _ | **1.3** |
| *Wy. confusa* | _ | _ | 1.3 | _ | _ | _ | _ | 4.0 | _ | _ | _ | 1.3 | _ | _ | 5.0 | _ | 93.3 | 10.0 | _ | _ | _ | _ | _ | 20.0 | _ | _ | **134.8** |
| *Wy. davisi* | _ | _ | _ | _ | _ | 3.3 | _ | _ | _ | _ | 0.4 | 1.3 | _ | _ | _ | _ | _ | _ | _ | _ | _ | _ | _ | 30.0 | 8.0 | _ | **43.0** |
| *Wy. edwardsi* | _ | 8.3 | _ | _ | _ | 0.8 | _ | _ | _ | _ | _ | _ | 20.0 | _ | _ | _ | _ | _ | _ | 0.8 | _ | _ | _ | _ | _ | _ | **30.0** |
| *Wy. exallos'* | _ | _ | _ | _ | _ | _ | _ | 1.0 | _ | _ | _ | _ | _ | _ | _ | _ | _ | _ | _ | _ | _ | _ | _ | _ | _ | _ | **1.0** |
| *Wy. incaudata* | _ | 13.3 | _ | _ | _ | _ | _ | 2.0 | _ | _ | _ | _ | 2.5 | _ | _ | _ | _ | _ | _ | _ | _ | _ | _ | _ | _ | _ | **17.8** |
| *Wy. longirostris* | _ | _ | _ | _ | _ | _ | _ | 1.0 | _ | _ | _ | _ | _ | _ | _ | _ | _ | _ | _ | _ | _ | _ | _ | 10.0 | _ | _ | **11.0** |
| *Wy. lutzi* | _ | 1.7 | _ | _ | _ | _ | _ | _ | _ | _ | 0.8 | _ | _ | _ | _ | _ | _ | _ | _ | _ | _ | _ | _ | 20.0 | _ | _ | **22.4** |
| *Wy. medioalbipes* | _ | _ | 7.5 | _ | _ | _ | _ | _ | _ | _ | _ | _ | _ | _ | _ | _ | _ | _ | _ | _ | _ | _ | _ | _ | _ | _ | **7.5** |
| *Wy. melanocephala* | _ | _ | _ | _ | _ | _ | _ | _ | _ | _ | _ | _ | _ | _ | _ | _ | _ | _ | _ | _ | _ | _ | 1.3 | _ | _ | _ | **1.3** |
| *Wy. mystes* | _ | _ | _ | 40.0 | _ | 3.3 | _ | _ | _ | _ | _ | _ | 2.5 | _ | _ | _ | _ | _ | _ | 0.8 | _ | _ | _ | _ | 1.0 | _ | **47.7** |
| *Wy. oblita* | _ | 3.3 | _ | _ | _ | _ | _ | _ | _ | _ | _ | _ | _ | _ | _ | _ | _ | _ | _ | _ | _ | _ | _ | _ | _ | _ | **3.3** |
| *Wy. pallidoventer* | _ | _ | _ | _ | _ | _ | _ | _ | _ | _ | _ | 7.5 | _ | _ | _ | _ | _ | _ | _ | _ | _ | _ | _ | _ | _ | _ | **7.5** |
| *Wy. palmata/galvaoi* | _ | 30.0 | _ | 80.0 | _ | _ | _ | _ | _ | _ | _ | 3.8 | _ | _ | _ | _ | 1.7 | _ | _ | _ | _ | _ | _ | 50.0 | _ | _ | **165.4** |
| *Wy. pilicauda* | _ | _ | _ | 160.0 | _ | 11.7 | 5.0 | _ | _ | _ | 1.5 | 10.0 | 10.0 | _ | _ | _ | 8.3 | _ | _ | 3.3 | _ | _ | 1.3 | 30.0 | 29.0 | _ | **270.1** |
| *Wy. theobaldi* | _ | _ | _ | _ | _ | 2.5 | _ | _ | _ | _ | _ | _ | _ | _ | _ | _ | _ | _ | _ | _ | _ | _ | _ | _ | 1.0 | _ | **3.5** |
| *Wyeomyia sp.* | _ | 6.7 | 1.9 | 20.0 | 20.0 | 16.7 | 20.0 | 1.0 | 65.5 | 5.0 | 10.0 | 42.5 | 2.5 | 90.0 | 7.5 | 140.0 | 20.0 | 12.5 | 2.5 | 0.8 | _ | _ | _ | _ | 9.0 | _ | **494.0** |
| **Nº total** | **13** | **151.7** | **510.6** | **930** | **420** | **303** | **98** | **113** | **143.6** | **80** | **330.8** | **401.3** | **148** | **720** | **235** | **560** | **173.3** | **63** | **210** | **503.3** | **87.5** | **1280** | **566.3** | **310** | **142** | **40** | **8531** |

1: Density was calculated by dividing the number of mosquitoes collected per person per day x 10. We marked with ‘ those taxa with ambiguous classification, due to the existence of complex of cryptic species.
