# Supplementary material for: Haemagogus leucocelaenus and Haemagogus janthinomys are the primary vectors in the major yellow fever outbreak in Brazil, 2016–2018
Source: Emerg Microbes Infect. 2019 Feb 1;8(1):218–31. doi: 10.1080/22221751.2019.1568180 (PMC6455131; doi:10.1080/22221751.2019.1568180)
Supplement: Supplemental Material [file TEMI_A_1568180_SM4764.zip › Table_Suppl_2_RLO.docx]

Supplementary table 2: Density of mosquito species^1^ captured during the YFV outbreak in each municipality. Dates of collections are in parenthesis: AR: Angra dos Reis (Fev 2018), BB: Belmiro Braga (Jan 2018), CR: Carmo (Apr 2017) CA: Casimiro de Abreu (Mar, May and Jun 2017), DM: Domingos Martins (Fev 2017), IB: Ibatiba (Fev 2017), IT: Itatiaia (May 2018), JF: Juiz de Fora (Jan 2018), MC: Macaé (Apr 2017), MT: Mangaratiba (Apr 2018), MH: Manhumirim (Fev 2017), MR: Maricá (May 2017), NT: Niterói (Jan 2018), SV: Salvador (Jun 2017), SE: Serra (Mar 2017), SM: Simonésia (Fev 2017), TR: Teresópolis (Dec 2017 and Fev 2018), VL: Valença (Jan-Fev 2018), VI: Vitória (Mar 2017), VR: Volta Redonda (Mar and May 2018).

|  | **AR** | **BB** | **CR** | **CA** | **DM** | **IB** | **IT** | **JF** | **MC** | **MT** | **MH** | **MR** | **NT** | **NI** | **SV** | **SE** | **SM** | **TR** | **VL** | **VI** | **VR** | **TOTAL** | |
| --- | --- | --- | --- | --- | --- | --- | --- | --- | --- | --- | --- | --- | --- | --- | --- | --- | --- | --- | --- | --- | --- | --- | --- |
| **Human cases** | 57 | 1 | 0 | 8 | 25 | 23 | 5 | 43 | 5 | 1 | 8 | 3 | 0 | 0 | 0 | 1 | 12 | 23 | 40 | 0 | 0 | **_** | |
| **Confirmed pools** | 1 | 2 | 0 | 0 | 2 | 0 | 0 | 1 | 1 | 0 | 0 | 39 | 0 | 1 | 0 | 0 | 0 | 2 | 15 | 0 | 0 | **_** | |
| *Ae. aegypti* | _ | _ | _ | _ | _ | _ | _ | _ | _ | _ | _ | 0.04 | _ | _ | _ | 0.1 | _ | 1.0 | 3.9 | 2.5 | 26.7 | **34** |  |
| *Ae. albopictus* | 60.0 | 27.5 | 25.0 | 2.2 | 0.6 | _ | 2.5 | 140.0 | 1.7 | _ | 10.0 | 0.8 | 77.5 | 6.7 | 10.2 | 1.3 | 5.8 | 3.0 | 5.1 | 40.0 | 33.3 | **453** |  |
| *Ae. argyrothorax* | _ | _ | _ | _ | 0.1 | _ | _ | _ | _ | _ | _ | 0.04 | _ | _ | _ | _ | _ | _ | _ | _ | _ | **0** |  |
| *Ae. condolences'* | _ | _ | _ | _ | _ | _ | _ | 20.0 | _ | _ | _ | _ | _ | _ | _ | _ | _ | _ | _ | _ | _ | **20** |  |
| *Ae. fluviatilis* | _ | _ | _ | _ | _ | _ | _ | 40.0 | _ | _ | _ | _ | _ | _ | _ | _ | _ | 2.0 | _ | _ | _ | **42** |  |
| *Ae. fulvithorax* | _ | _ | _ | 0.1 | _ | _ | _ | _ | _ | _ | _ | _ | _ | _ | _ | _ | _ | _ | _ | _ | _ | **0** |  |
| *Ae. rhyacophilus* | _ | _ | _ | _ | 0.1 | 5.0 | _ | _ | 1.7 | _ | _ | _ | _ | _ | _ | _ | _ | _ | _ | _ | _ | **7** |  |
| *Ae. scapularis* | 13.3 | 12.5 | 35.0 | 14.6 | 3.5 | 10.0 | _ | 5.0 | 45.0 | _ | 80.0 | 30.9 | _ | 6.7 | 1.0 | 21.9 | 6.3 | 14.0 | 1.5 | 10.6 | 1.7 | **313** |  |
| *Ae. serratus* | 23.3 | 102.5 | _ | 0.0 | 0.8 | 5.0 | _ | 5.0 | 1.7 | _ | _ | _ | _ | _ | _ | _ | _ | 1.0 | 0.2 | _ | _ | **139** |  |
| *Ae. taeniorhynchus* | 3.3 | _ | _ | _ | _ | _ | _ | _ | _ | 5.0 | _ | 77.5 | _ | _ | _ | 1.1 | _ | _ | _ | 285.6 | _ | **373** |  |
| *Ae. terrens* | 1.7 | 2.5 | _ | 0.1 | _ | _ | _ | _ | 11.7 | _ | _ | _ | _ | _ | _ | _ | 0.6 | 8.0 | 0.1 | _ | _ | **25** |  |
| *Aedeomyia sp.* | _ | _ | _ | 0.0 | _ | _ | _ | _ | _ | _ | _ | _ | _ | _ | _ | _ | _ | _ | _ | _ | _ | **0** |  |
| *Aedes sp.* | _ | 5.0 | _ | 0.1 | 0.1 | _ | _ | _ | _ | _ | _ | 0.6 | _ | _ | 0.4 | 0.1 | 1.0 | 1.0 | 0.1 | 21.9 | _ | **30** |  |
| *Anopheles sp.* | _ | _ | _ | _ | _ | _ | _ | _ | _ | _ | _ | _ | _ | _ | _ | _ | 0.1 | _ | _ | _ | _ | **0** |  |
| *Coquillettidia sp.* | _ | _ | _ | _ | _ | _ | _ | _ | _ | _ | _ | _ | _ | _ | _ | 4.6 | 1.9 | _ | 0.3 | _ | _ | **7** |  |
| *Cq. albicosta* | _ | _ | _ | _ | _ | _ | _ | _ | _ | _ | _ | _ | _ | _ | _ | _ | 0.1 | _ | 0.4 | _ | _ | **1** |  |
| *Cq. hermanoi'* | _ | _ | _ | _ | _ | _ | _ | _ | _ | _ | _ | _ | _ | _ | _ | 0.1 | _ | _ | _ | _ | _ | **0** |  |
| *Cq. justamansonia* | _ | _ | 5.0 | _ | 0.1 | _ | _ | _ | _ | _ | _ | _ | _ | _ | _ | 1.0 | _ | _ | _ | _ | _ | **6** |  |
| *Cq. nigricans* | _ | _ | _ | _ | _ | _ | _ | _ | _ | _ | _ | _ | _ | _ | _ | 0.6 | _ | _ | _ | _ | _ | **1** |  |
| *Cq. shannoni* | _ | _ | _ | _ | _ | _ | _ | _ | _ | _ | _ | _ | _ | _ | _ | 0.1 | _ | _ | _ | _ | _ | **0** |  |
| *Cq. venezuelensis* | _ | _ | _ | _ | _ | _ | _ | _ | _ | _ | _ | _ | _ | _ | _ | _ | 0.7 | _ | 0.5 | _ | _ | **1** |  |
| *Culex coronator'* | _ | _ | _ | _ | _ | 10.0 | _ | _ | _ | _ | _ | _ | _ | _ | _ | _ | _ | _ | _ | _ | _ | **10** |  |
| *Culex sp.* | _ | _ | _ | 19.0 | 0.2 | 60.0 | _ | _ | _ | _ | 40.0 | 6.3 | _ | _ | _ | 4.8 | 1.4 | 31.0 | 1.0 | 1.9 | _ | **166** |  |
| *Culicidae* | _ | _ | _ | 0.4 | _ | _ | _ | _ | _ | _ | _ | _ | _ | _ | _ | _ | _ | _ | _ | _ | _ | **0** |  |
| *Cx. declarator'* | _ | _ | _ | _ | _ | _ | _ | _ | _ | _ | _ | 0.2 | _ | _ | _ | 0.1 | _ | _ | _ | _ | _ | **0** |  |
| *Cx. nigripalpus* | _ | _ | _ | 0.0 | 0.2 | _ | _ | _ | _ | _ | 10.0 | 5.2 | _ | _ | _ | 0.1 | _ | _ | 0.3 | _ | _ | **16** |  |
| *Cx. quinquefasciatus* | _ | _ | _ | 0.1 | _ | _ | _ | _ | _ | _ | _ | _ | _ | _ | _ | 0.3 | _ | _ | _ | 0.6 | 35.0 | **36** |  |
| *Hg. janthinomys* | _ | 190.0 | 5.0 | 1.2 | 0.1 | _ | _ | 95.0 | 28.3 | _ | 5.0 | 0.6 | _ | 56.7 | _ | _ | 5.3 | 76.0 | 21.4 | _ | _ | **484** |  |
| *Hg. leucocelaenus* | _ | 185.0 | 5.0 | 1.0 | 1.0 | _ | 5.0 | 65.0 | 40.0 | 5.0 | _ | 16.9 | _ | 35.0 | _ | _ | 9.7 | 55.0 | 15.8 | 0.6 | _ | **440** |  |
| *Li. durhamii* | 10.0 | 32.5 | 10.0 | 1.8 | 3.0 | _ | 2.5 | 75.0 | 5.0 | 5.0 | 15.0 | 2.9 | _ | 3.3 | _ | _ | 15.6 | 18.0 | 2.1 | 0.6 | _ | **202** |  |
| *Li. flavisetosus* | 1.7 | _ | _ | _ | _ | _ | _ | _ | _ | 5.0 | _ | _ | _ | _ | _ | _ | _ | 5.0 | _ | _ | _ | **12** |  |
| *Li. pseudomethisticus* | 1.7 | _ | _ | 0.2 | 0.2 | _ | 2.5 | _ | _ | 10.0 | _ | _ | _ | _ | _ | _ | _ | _ | _ | _ | _ | **15** |  |
| *Limatus sp.* | _ | _ | _ | 1.5 | 0.1 | _ | _ | _ | _ | _ | _ | 0.1 | _ | _ | _ | _ | 1.0 | _ | _ | _ | _ | **3** |  |
| *Lutzia sp.'* | _ | _ | _ | 0.1 | _ | _ | _ | _ | _ | _ | _ | _ | _ | _ | _ | _ | _ | _ | _ | _ | _ | **0** |  |
| *Ma. indubitans* | _ | _ | _ | _ | _ | _ | _ | _ | _ | _ | _ | _ | _ | _ | _ | 5.6 | _ | _ | _ | _ | _ | **6** |  |
| *Ma. titillans* | _ | _ | _ | 0.0 | _ | _ | _ | _ | _ | _ | _ | 0.1 | _ | _ | _ | 1.1 | _ | _ | _ | _ | _ | **1** |  |
| *Mansonia sp.* | _ | _ | _ | 0.1 | _ | _ | _ | _ | _ | _ | _ | _ | _ | _ | _ | 0.7 | _ | _ | _ | _ | _ | **1** |  |
| *On. personatum* | _ | 2.5 | _ | _ | 0.1 | _ | _ | _ | 3.3 | 15.0 | _ | _ | _ | 1.7 | _ | _ | _ | 9.0 | _ | _ | _ | **32** |  |
| *Ps. albipes* | _ | _ | _ | 0.0 | _ | _ | _ | _ | _ | _ | _ | _ | _ | _ | _ | _ | _ | _ | _ | _ | _ | **0** |  |
| *Ps. ferox* | 30.0 | 157.5 | _ | 0.1 | 0.3 | _ | _ | 10.0 | _ | _ | 15.0 | _ | _ | 8.3 | _ | 0.1 | 2.4 | 1.0 | 0.4 | 0.6 | _ | **226** |  |
| *Ps. lutzii/amazonica* | 6.7 | _ | _ | _ | 0.1 | _ | _ | _ | _ | _ | _ | _ | _ | _ | _ | _ | _ | _ | _ | _ | _ | **7** |  |
| *Psorophora sp.* | 10.0 | 7.5 | _ | 0.1 | 0.5 | _ | _ | _ | _ | _ | 10.0 | _ | _ | _ | _ | 0.1 | 0.4 | _ | _ | _ | _ | **29** |  |
| *Ru. cerqueirai* | _ | _ | _ | _ | _ | _ | _ | _ | _ | _ | _ | _ | _ | _ | _ | 0.1 | _ | 2.0 | _ | 1.3 | _ | **3** |  |
| *Ru. frontosa* | 1.7 | _ | _ | 0.3 | _ | _ | _ | _ | _ | 5.0 | _ | 0.1 | _ | _ | _ | _ | _ | _ | _ | 1.3 | _ | **8** |  |
| *Ru. humboldti* | _ | _ | _ | _ | 0.1 | _ | _ | _ | 1.7 | _ | _ | 0.4 | _ | _ | _ | _ | _ | _ | 0.1 | _ | _ | **2** |  |
| *Runchomyia sp.* | 3.3 | _ | _ | 0.1 | 0.1 | _ | 2.5 | _ | _ | _ | _ | 0.3 | _ | _ | _ | _ | 0.7 | 1.0 | _ | _ | _ | **8** |  |
| *Sa. albiprivus* | 3.3 | _ | 95.0 | 0.2 | 0.1 | _ | _ | _ | 11.7 | _ | _ | _ | _ | _ | _ | 0.1 | 35.4 | 1.0 | _ | 5.0 | _ | **152** |  |
| *Sa. aurescens* | _ | _ | _ | 0.1 | _ | _ | _ | _ | _ | _ | _ | _ | _ | _ | _ | _ | _ | 5.0 | _ | _ | _ | **5** |  |
| *Sa. chloropterus* | 1.7 | _ | _ | 0.3 | 0.2 | _ | _ | _ | _ | _ | _ | 0.2 | _ | 15.0 | _ | _ | 0.4 | _ | _ | _ | _ | **18** |  |
| *Sa. fabricii/undosus* | 13.3 | _ | _ | 0.1 | 0.1 | _ | _ | _ | _ | _ | 5.0 | _ | _ | _ | _ | _ | _ | 32.0 | 0.1 | _ | _ | **51** |  |
| *Sa. identicus* | 1.7 | _ | 10.0 | 0.0 | _ | _ | _ | _ | _ | _ | _ | _ | _ | 5.0 | _ | _ | 0.3 | 1.0 | _ | _ | _ | **18** |  |
| *Sa. intermedius* | 3.3 | _ | _ | _ | _ | _ | _ | _ | _ | _ | _ | _ | _ | _ | _ | _ | _ | 1.0 | _ | _ | _ | **4** |  |
| *Sa. melanonymphe* | 1.7 | _ | _ | _ | _ | _ | 2.5 | _ | _ | _ | _ | _ | _ | 1.7 | _ | _ | 0.1 | 1.0 | _ | _ | _ | **7** |  |
| *Sa. petrocchiae'* | _ | _ | _ | _ | _ | _ | _ | _ | _ | _ | _ | _ | _ | _ | _ | _ | 24.7 | _ | _ | _ | _ | **25** |  |
| *Sa. purpureus'* | _ | _ | _ | _ | _ | _ | 15.0 | _ | _ | _ | _ | _ | _ | _ | _ | _ | _ | _ | 0.1 | _ | _ | **15** |  |
| *Sa. quasicyaneus* | _ | _ | _ | _ | _ | _ | _ | _ | _ | _ | _ | _ | _ | _ | _ | _ | 0.1 | _ | _ | _ | _ | **0** |  |
| *Sa. soperi* | 1.7 | _ | _ | _ | _ | _ | _ | _ | _ | _ | _ | _ | _ | _ | _ | _ | _ | _ | 0.2 | _ | _ | **2** |  |
| *Sa. whitmani* | _ | _ | _ | _ | 0.1 | _ | _ | _ | _ | _ | _ | _ | _ | _ | _ | _ | 0.4 | _ | _ | _ | _ | **1** |  |
| *Sa. xyphydes* | _ | _ | _ | 0.03 | _ | _ | _ | _ | _ | _ | _ | _ | _ | 1.7 | _ | _ | _ | _ | 0.1 | _ | _ | **2** |  |
| *Sabethes sp.* | 6.7 | _ | _ | 0.2 | 0.2 | _ | _ | _ | _ | _ | _ | _ | _ | 1.7 | 0.2 | _ | 5.4 | _ | 0.2 | _ | _ | **15** |  |
| *Sh. fluviatilis* | _ | _ | _ | _ | 0.2 | _ | _ | _ | 3.3 | _ | _ | _ | _ | _ | _ | _ | _ | 28.0 | _ | _ | _ | **32** |  |
| *Shannoniana sp.* | _ | _ | _ | _ | _ | _ | _ | _ | _ | _ | _ | _ | _ | _ | _ | _ | _ | 1.0 | _ | _ | _ | **1** |  |
| *Tr. castroi/similis* | _ | _ | _ | 0.0 | _ | _ | _ | _ | _ | _ | _ | _ | _ | _ | _ | _ | _ | _ | _ | _ | _ | **0** |  |
| *Tr. compressum* | 1.7 | _ | _ | 0.2 | _ | _ | _ | _ | _ | _ | _ | _ | _ | _ | _ | _ | _ | 4.0 | _ | _ | _ | **6** |  |
| *Tr. digitatum* | _ | 2.5 | _ | 0.1 | 0.1 | _ | _ | 5.0 | 1.7 | _ | _ | 0.0 | _ | _ | _ | _ | _ | 6.0 | _ | _ | _ | **15** |  |
| *Tr. pallidiventer* | _ | 2.5 | _ | 0.0 | 0.9 | _ | _ | 15.0 | _ | 5.0 | _ | _ | _ | 5.0 | _ | _ | _ | 13.0 | 0.1 | _ | _ | **42** |  |
| *Tr. soaresi* | 1.7 | _ | _ | _ | _ | _ | _ | _ | _ | _ | _ | _ | _ | _ | _ | _ | 0.1 | _ | _ | _ | _ | **2** |  |
| *Trichoprosopon sp.* | 1.7 | _ | _ | 0.3 | 0.1 | _ | 2.5 | _ | _ | _ | _ | 0.1 | _ | _ | _ | _ | 0.3 | 1.0 | _ | _ | _ | **6** |  |
| *Wy. aporonoma/staminifera* | _ | 45.0 | _ | 0.5 | 0.2 | _ | 2.5 | 45.0 | 1.7 | 15.0 | _ | _ | _ | 13.3 | _ | _ | 1.1 | 10.0 | 0.2 | _ | _ | **135** |  |
| *Wy. arthrostigma'* | _ | _ | _ | _ | _ | _ | _ | _ | _ | _ | _ | _ | _ | _ | _ | _ | 0.1 | _ | _ | _ | _ | **0** |  |
| *Wy. bonnei/deanei* | _ | _ | _ | 0.1 | 0.1 | _ | _ | _ | _ | _ | _ | _ | _ | _ | _ | _ | _ | 3.0 | _ | _ | _ | **3** |  |
| *Wy. bourrouli/ forcipenis* | _ | _ | 20.0 | _ | 0.2 | _ | _ | 5.0 | _ | _ | _ | 0.2 | _ | 1.7 | _ | _ | _ | _ | _ | _ | _ | **27** |  |
| *Wy. codiocampa* | _ | _ | _ | _ | 0.1 | _ | _ | _ | _ | _ | _ | _ | _ | _ | _ | _ | _ | _ | _ | _ | _ | **0** |  |
| *Wy. confusa* | _ | _ | _ | 1.1 | 1.1 | _ | _ | 5.0 | 3.3 | _ | _ | 0.5 | _ | _ | _ | _ | 0.6 | 40.0 | 0.3 | _ | _ | **52** |  |
| *Wy. davisi* | _ | _ | _ | _ | _ | _ | _ | _ | _ | 65.0 | _ | _ | _ | _ | _ | _ | _ | _ | _ | _ | _ | **65** |  |
| *Wy. dyari* | _ | _ | _ | 0.03 | _ | _ | _ | _ | _ | _ | _ | _ | _ | 6.7 | _ | _ | _ | _ | _ | _ | _ | **7** |  |
| *Wy. edwardsi* | _ | _ | 10.0 | _ | 0.1 | _ | _ | _ | _ | _ | _ | _ | _ | 1.7 | _ | _ | _ | _ | _ | _ | _ | **12** |  |
| *Wy. incaudata* | _ | _ | _ | 0.03 | _ | _ | _ | _ | _ | _ | _ | _ | _ | 11.7 | _ | _ | _ | 2.0 | _ | _ | _ | **14** |  |
| *Wy. knabi'* | _ | _ | _ | _ | 0.1 | _ | _ | _ | _ | _ | _ | _ | _ | _ | _ | _ | _ | _ | _ | _ | _ | **0** |  |
| *Wy. lutzi* | 5.0 | _ | _ | _ | _ | 5.0 | _ | _ | _ | _ | _ | _ | _ | _ | _ | _ | _ | 7.0 | 0.1 | _ | _ | **17** |  |
| *Wy. medioalbipes* | _ | _ | 80.0 | 0.03 | 0.1 | _ | _ | _ | _ | _ | _ | _ | _ | _ | _ | _ | 2.8 | _ | 0.3 | _ | _ | **83** |  |
| *Wy. melanocephala* | _ | _ | _ | _ | _ | _ | _ | _ | _ | _ | _ | _ | _ | _ | _ | _ | 0.1 | _ | _ | _ | _ | **0** |  |
| *Wy. mystes* | 5.0 | _ | _ | 0.2 | 0.4 | _ | _ | _ | 5.0 | _ | _ | 0.2 | _ | _ | _ | _ | 0.6 | 3.0 | 0.1 | _ | _ | **14** |  |
| *Wy. oblita* | _ | _ | _ | _ | _ | _ | _ | _ | _ | _ | _ | _ | _ | _ | _ | _ | _ | 1.0 | _ | _ | _ | **1** |  |
| *Wy. palmata/galvaoi* | 1.7 | _ | _ | _ | _ | _ | _ | _ | _ | _ | _ | _ | _ | 28.3 | _ | _ | _ | 6.0 | _ | _ | _ | **36** |  |
| *Wy. pilicauda* | 1.7 | _ | 5.0 | 0.1 | _ | _ | _ | _ | _ | _ | _ | _ | _ | 35.0 | _ | _ | _ | 15.0 | _ | _ | _ | **57** |  |
| *Wy. shannoni* | _ | _ | _ | _ | _ | _ | _ | _ | _ | _ | _ | _ | _ | _ | _ | _ | 0.1 | _ | _ | _ | _ | **0** |  |
| *Wyeomyia sp.* | 16.7 | _ | 10.0 | 1.8 | 0.6 | _ | 10.0 | _ | _ | 30.0 | 15.0 | 0.3 | _ | 15.0 | _ | _ | 5.6 | 73.0 | 0.2 | 1.9 | _ | **180** |  |
| **Nº total** | **235** | **775** | **315** | **49** | **16** | **95** | **48** | **530** | **167** | **165** | **205** | **144** | **78** | **262** | **12** | **44** | **131** | **482** | **55** | **374** | **97** | **4278** | |

1: Density is calculated by dividing the number of mosquitoes collected per person and/or trap per day x 10. We marked with ‘ those taxa with ambiguous classification, due to the existence of complex of cryptic species.
