# Supplementary material for: Haemagogus leucocelaenus and Haemagogus janthinomys are the primary vectors in the major yellow fever outbreak in Brazil, 2016–2018
Source: Emerg Microbes Infect. 2019 Feb 1;8(1):218–31. doi: 10.1080/22221751.2019.1568180 (PMC6455131; doi:10.1080/22221751.2019.1568180)
Supplement: Supplemental Material [file TEMI_A_1568180_SM4764.zip › Table_Suppl_3_RLO.docx]

Supplementary table 3: Detailed list of infected pools with CT-values. Confirmation of YF diagnosis was done by amplifying and sequencing the 650bp amplicon, following edition of nucleic acid sequences and comparison with other YFV strains available at the Genbank database using BLAST.

| ***State*** | ***Municipality*** | ***Collection date*** | ***Code*** | ***Species*** | ***Nº*** | **CT mean** |
| --- | --- | --- | --- | --- | --- | --- |
| ES | Domingos Martins | 23/02/2017 | PA 193^1^ | *Hg. leucocelaenus* | 5 | RT-PCR^3^ |
| ES | Domingos Martins | 23/02/2017 | PA 196^2^ | *Hg. janthinomys* | 1 | RT-PCR^3^ |
| RJ | Macaé | 26/04/2017 | MC 941 | *Hg. janthinomys* | 2 | 22.6 |
| RJ | Maricá | 04/05/2017 | MR 949 | *Hg. leucocelaenus* | 5 | 21.1 |
| RJ | Maricá | 04/05/2017 | MR 951 | *Hg. leucocelaenus* | 5 | 19.7 |
| RJ | Maricá | 08/05/2017 | MR 960 | *Hg. janthinomys* | 1 | 30.0 |
| RJ | Maricá | 08/05/2017 | MR 961 | *Hg. leucocelaenus* | 6 | 22.4 |
| RJ | Maricá | 08/05/2017 | MR 963 | *Hg. leucocelaenus* | 5 | 19.4 |
| RJ | Maricá | 08/05/2017 | MR 964 | *Hg. leucocelaenus* | 5 | 20.3 |
| RJ | Maricá | 08/05/2017 | MR 965 | *Hg. leucocelaenus* | 5 | 17.0 |
| RJ | Maricá | 08/05/2017 | MR 967 | *Hg. leucocelaenus* | 5 | 18.2 |
| RJ | Maricá | 08/05/2017 | MR 968 | *Hg. leucocelaenus* | 5 | 19.0 |
| RJ | Maricá | 05/05/2017 | MR 970 | *Hg. leucocelaenus* | 5 | 18.7 |
| RJ | Maricá | 05/05/2017 | MR 971 | *Hg. leucocelaenus* | 5 | 18.7 |
| RJ | Maricá | 05/05/2017 | MR 972 | *Hg. leucocelaenus* | 5 | 19.7 |
| RJ | Maricá | 05/05/2017 | MR 973 | *Hg. leucocelaenus* | 5 | 36.2 |
| RJ | Maricá | 05/05/2017 | MR 974 | *Hg. leucocelaenus* | 2 | 18.1 |
| RJ | Maricá | 05/05/2017 | MR 976 | *Hg. janthinomys* | 6 | 19.0 |
| RJ | Maricá | 05/05/2017 | MR 977 | *Hg. leucocelaenus* | 5 | 17.2 |
| RJ | Maricá | 05/05/2017 | MR 978 | *Hg. leucocelaenus* | 5 | 16.1 |
| RJ | Maricá | 05/05/2017 | MR 980 | *Hg. leucocelaenus* | 6 | 18.9 |
| RJ | Maricá | 05/05/2017 | MR 981 | *Hg. leucocelaenus* | 5 | 19.0 |
| RJ | Maricá | 06/05/2017 | MR 984 | *Hg. leucocelaenus* | 2 | 21.2 |
| RJ | Maricá | 08/05/2017 | MR 1035 | *Hg. leucocelaenus* | 4 | 22.4 |
| RJ | Maricá | 08/05/2017 | MR 1044 | *Hg. leucocelaenus* | 5 | 10.5 |
| RJ | Maricá | 08/05/2017 | MR 1094 | *Hg. leucocelaenus* | 9 | 18.6 |
| RJ | Maricá | 05/05/2017 | MR 1112 | *Hg. leucocelaenus* | 4 | 11.2 |
| RJ | Maricá | 05/05/2017 | MR 1139 | *Hg. leucocelaenus* | 10 | 10.0 |
| RJ | Maricá | 05/05/2017 | MR 1140 | *Hg. leucocelaenus* | 10 | 11.6 |
| RJ | Maricá | 05/05/2017 | MR 1145 | *Hg. leucocelaenus* | 10 | 11.2 |
| RJ | Maricá | 05/05/2017 | MR 1152 | *Hg. leucocelaenus* | 11 | 10.7 |
| RJ | Maricá | 05/05/2017 | MR 1158 | *Hg. leucocelaenus* | 5 | 11.8 |
| RJ | Maricá | 05/05/2017 | MR 1160 | *Hg. leucocelaenus* | 5 | 12.8 |
| RJ | Maricá | 06/05/2017 | MR 1351 | *Hg. leucocelaenus* | 4 | 12.5 |
| RJ | Maricá | 06/05/2017 | MR 1353 | *Ae. scapularis* | 8 | 23.0 |
| RJ | Maricá | 06/05/2017 | MR 1356 | *Ae. taeniorhynchus* | 11 | 21.9 |
| RJ | Maricá | 07/05/2017 | MR 1358 | *Hg. leucocelaenus* | 2 | 12.0 |
| RJ | Maricá | 07/05/2017 | MR 1391 | *Hg. leucocelaenus* | 2 | 24.6 |
| RJ | Maricá | 04/05/2017 | MR 1438 | *Hg. leucocelaenus* | 3 | 15.6 |
| RJ | Maricá | 05/05/2017 | PFA 121 | *Hg. leucocelaenus* | 9 | 17.4 |
| RJ | Maricá | 05/05/2017 | PFA122 | *Hg. leucocelaenus* | 6 | 18.3 |
| RJ | Maricá | 05/05/2017 | PFA123 | *Hg. leucocelaenus* | 10 | 18.4 |
| RJ | Teresópolis | 19/12/2017 | TR 2807 | *Hg. janthinomys* | 5 | 31.0 |
| RJ | Teresópolis | 19/12/2017 | TR 2848 | *Hg. leucocelaenus* | 2 | 32.1 |
| RJ | Nova Iguaçú | 09/01/2018 | NI 3104 | *Hg. janthinomys* | 5 | 17.2 |
| RJ | Valença | 18/01/2018 | VL 2909 | *Hg. janthinomys* | 5 | 21.7 |
| RJ | Valença | 19/01/2018 | VL 2926 | *Hg. janthinomys* | 1 | 17.5 |
| RJ | Valença | 24/01/2018 | VL 2953 | *Hg. janthinomys* | 4 | 24.0 |
| RJ | Valença | 24/01/2018 | VL 2964 | *Hg. janthinomys* | 5 | 25.0 |
| RJ | Valença | 24/01/2018 | VL 2967 | *Hg. janthinomys* | 5 | 16.9 |
| RJ | Valença | 24/01/2018 | VL 2969 | *Hg. leucocelaenus* | 3 | 18.7 |
| RJ | Valença | 24/01/2018 | VL 3003 | *Hg. janthinomys* | 4 | 28.3 |
| RJ | Valença | 24/01/2018 | VL 3005 | *Hg. janthinomys* | 5 | 24.6 |
| RJ | Valença | 24/01/2018 | VL 3006 | *Hg. janthinomys* | 4 | 18.2 |
| RJ | Valença | 26/01/2018 | VL 3012 | *Hg. janthinomys* | 5 | 19.2 |
| RJ | Valença | 26/01/2018 | VL 3016 | *Hg. janthinomys* | 5 | 16.5 |
| RJ | Valença | 26/01/2018 | VL 3877 | *Hg. janthinomys* | 5 | 19.8 |
| RJ | Valença | 26/01/2018 | VL 3882 | *Hg. leucocelaenus* | 5 | 19.9 |
| RJ | Valença | 26/01/2018 | VL 3902 | *Hg. leucocelaenus* | 4 | 18.8 |
| RJ | Valença | 26/01/2018 | PFA131 | *Hg. janthinomys* | 10 | 18.5 |
| RJ | Angra dos Reis | 07/02/2018 | IG 3036 | *Sa. chloropterus* | 1 | 31.0 |
| MG | Juiz de Fora | 27/01/2018 | JF 2793 | *Hg. janthinomys* | 3 | RT-PCR^3^ |
| MG | Belmiro Braga | 18/01/2018 | MG 3121 | *Hg. leucocelaenus* | 3 | 16.4 |
| MG | Belmiro Braga | 29/01/2018 | MG 3155 | *Hg. janthinomys* | 5 | 16.1 |

^1^Complete genome deposited in Genbank (Nº MF423373.1); ^2^Complete genome deposited in Genbank (Nº MF423374.1); ^3^Sample tested by conventional RT-PCR.
